# Gene expression analyses reveal differences in children’s response to malaria according to their age

**DOI:** 10.1038/s41467-024-46416-3

**Published:** 2024-03-06

**Authors:** Kieran Tebben, Salif Yirampo, Drissa Coulibaly, Abdoulaye K. Koné, Matthew B. Laurens, Emily M. Stucke, Ahmadou Dembélé, Youssouf Tolo, Karim Traoré, Amadou Niangaly, Andrea A. Berry, Bourema Kouriba, Christopher V. Plowe, Ogobara K. Doumbo, Kirsten E. Lyke, Shannon Takala-Harrison, Mahamadou A. Thera, Mark A. Travassos, David Serre

**Affiliations:** 1 Institute for Genome Sciences, University of Maryland School of Medicine, Baltimore, MD USA; 2 Department of Microbiology and Immunology, University of Maryland School of Medicine, Baltimore, MD USA; 3 Malaria Research and Training Center, University of Sciences, Techniques and Technologies, Bamako, Mali; 4 Malaria Research Program, Center for Vaccine Development and Global Health, University of Maryland School of Medicine, Baltimore, MD USA

**Keywords:** Parasite host response, Malaria, Sequencing

## Abstract

In Bandiagara, Mali, children experience on average two clinical malaria episodes per year. However, even in the same transmission area, the number of uncomplicated symptomatic infections, and their parasitemia, can vary dramatically among children. We simultaneously characterize host and parasite gene expression profiles from 136 Malian children with symptomatic falciparum malaria and examine differences in the relative proportion of immune cells and parasite stages, as well as in gene expression, associated with infection and or patient characteristics. Parasitemia explains much of the variation in host and parasite gene expression, and infections with higher parasitemia display proportionally more neutrophils and fewer T cells, suggesting parasitemia-dependent neutrophil recruitment and/or T cell extravasation to secondary lymphoid organs. The child’s age also strongly correlates with variations in gene expression: *Plasmodium falciparum* genes associated with age suggest that older children carry more male gametocytes, while variations in host gene expression indicate a stronger innate response in younger children and stronger adaptive response in older children. These analyses highlight the variability in host responses and parasite regulation during *P. falciparum* symptomatic infections and emphasize the importance of considering the children’s age when studying and treating malaria infections.

## Abstract (web summary)

Here the authors use dual RNA sequencing to characterize host and parasite gene expression from 136 Malian children with symptomatic *Plasmodium falciparum* infection. They find that parasitemia levels correlate with neutrophil and T cell levels and that the child’s age correlates with innate immune gene expression as well as gametocyte levels.

## Introduction

Despite decades of progress towards elimination, malaria remains a major public health problem in endemic areas^1^. In 2021, there were 247 million cases of malaria worldwide, resulting in over 600,000 deaths, mostly in children younger than 5 years old^1^. Malaria is caused by *Plasmodium* parasites that are transmitted by *Anopheles* mosquitos^2^. While five *Plasmodium* species cause human malaria – *P. falciparum, P. vivax, P. ovale, P. malariae* and *P. knowlesi* – *P. falciparum* is responsible for most cases of malaria worldwide^2^ and causes the most severe forms of the disease^3^. All symptoms of malaria stem from the asexual replication of parasites in the blood, and therefore gene expression analysis of blood samples from infected patients can provide invaluable insights into the role of different host and parasite factors in regulating the disease. Several studies have previously used this approach to study human immune cells, revealing modulation of gene pathways regulated by pro-inflammatory cytokines over repeated malaria exposures in Malian adults^4^, and development of memory B cells with atypical gene expression patterns over repeated exposures^5^. Additionally, gene expression analysis has been used to study *Plasmodium* parasites, revealing parasitemia-dependent regulation of metabolism and cell death genes^6^, and coordination of *var* gene expression^7^. However, few studies have examined host and parasite transcripts from the same samples, and those have specifically focused on disease severity: one study identified unique host and parasite gene expression patterns associated with specific severe malaria complications (e.g., coma, hyperlactemia) and noted that human gene expression during severe malaria was driven by parasite load^8^. Another study identified differential activation of the innate immune system according to disease severity^9^. However, it remains unclear how gene expression varies among patients with uncomplicated malaria and whether these variations could explain some of the disease heterogeneity.

The response of the host immune system to *Plasmodium* parasites (and of the parasites to the host response) can occur both at the cellular and/or molecular level: specific white blood cell populations become activated upon infection and increase in their relative proportions, while some genes (in a given cell type) might be specifically up- or down-regulated in response to the infection. These two processes are difficult to disentangle in studies of gene expression performed from whole blood samples as the observed gene expression differences among samples could derive from both differential gene regulation of specific genes and variations, among patients, in the relative proportions of white blood cell subsets (or parasite developmental stages). Single cell RNA sequencing, or flow cytometry prior to bulk RNA sequencing (RNA-seq), can circumvent this issue, but these methods are difficult to implement in field settings and can be prohibitively expensive in studying large cohorts. Alternatively, one can computationally infer the proportions of different cells in a sample directly from bulk RNA-seq data using gene expression deconvolution^10^: by comparing the normalized gene expression of a bulk RNA-seq experiment with gene expression profiles of known cells, one can robustly estimate the relative proportions of both human immune cells^10–12^ and *Plasmodium* developmental stages (including gametocytes)^13–15^ present in a given blood sample, without the additional costs and resources required for scRNA-seq or flow cytometry, and thus obtain information on both the cell composition and gene expression.

Here, we used dual RNA-seq to simultaneously characterize host and parasite gene expression from 136 whole blood samples from Malian children during a symptomatic, uncomplicated *P. falciparum* infection. We first evaluated the contributions of different clinical and demographic parameters to the overall host and parasite gene expression profiles. We then used gene expression deconvolution to (i) estimate the relative proportions of the different immune cells and parasite developmental stages in each sample and assess their association with the child’s age and parasitemia, as well as to (ii) rigorously determine host and parasite genes whose expression were associated with these parameters.

## Results and discussion

### Comprehensive profiling of host and parasite transcriptomes by dual RNA sequencing

We analyzed 136 whole blood samples collected from children ages 1–15 years enrolled in a longitudinal incidence study in Bandiagara, Mali from 2009 – 2013^16^. All samples included here were collected during an uncomplicated, symptomatic *P. falciparum* malaria episode, defined by the study physicians as an unscheduled visit, initiated by the patient in response to malaria symptoms (fever, headaches, joint pain, vomiting, diarrhea, or abdominal pain), and with microscopic evidence of parasites^16^. Children of both sexes were included in roughly equal proportions and most participants were of Dogon ethnicity (Table 1). For a subset of 120 individuals with at least 2 years of follow-up, we also determined (i) the number of subsequent malaria episodes and (ii) the time to the next malaria episode (Table 1), and normalized these values by the monthly risk of malaria and the child’s total time in the study for our statistical analyses (see Materials and Methods for details). Values for all variables considered in this study, as well as the date of sample collection for each sample, are available in Supplementary Data 1.

**Table 1:** Characteristics of study participants

Variable		Median [range]
Participant’s age (years)		5 [1–15]
Parasitemia (parasites/µL)		26,675 [48–622,775]
No. of subsequent symptomatic malaria episodes^a^		5 [0–14]
Time to next symptomatic malaria episode (days)^a^		86.5 [140]

		Count (%)
Participant Ethnicity	Dogon	101 (74%)
	Other	35 (26%)
Complexity of Infection^b^	Monoclonal infection	49 (36%)
	Polyclonal infection	87 (64%)
Participant’s sex	Male	72 (53%)
	Female	64 (47%)

^a^Only the subset of participants with at least 2 years of follow-up after the sample collection were included for statistical analyses. Note that these values were subsequently normalized (by the monthly risk of malaria and the child’s total time in the study) for statistical analyses.

^b^Complexity of infection estimated from polymorphisms determined from the RNA-seq data.

From each whole blood sample (*N* = 136), we extracted and sequenced RNA to characterize the host and parasite gene expression profiles (Supplementary Data 1). To confirm that *P. falciparum* was responsible for each malaria episode and exclude possible co-infections, we first mapped all reads, simultaneously, to the genomes of *P. falciparum*, *P. malariae*, *P. ovale* and *P. vivax*. In each sample, 95% or more of the reads that mapped to a *Plasmodium* genome were uniquely mapped to *P. falciparum* and no co-infections were detected (Supplementary Data 2). (Note, that the small proportion of reads mapping to a species other than *P. falciparum* likely reflects reads derived from highly conserved regions that can be mapped to multiple species).

We then mapped all reads simultaneously to the *P. falciparum* and human genomes and obtained, on average, 85 million reads (21,121,310–149,135,348) mapped to the human genome (30.2–99.8%) and 14 million reads (160,712–64,001,204) mapped to the *P. falciparum* genome (0.2–69.8%) (Supplementary Data 1), allowing robust characterization of each transcriptome. As expected, the proportion of reads mapped to the *P. falciparum* genome was significantly correlated with the parasitemia determined microscopically (*p* = 6.03 × 10^−14^, *r*^2^ = 0.34, Supplementary Fig. 1). Overall, we were able to analyze variations in expression for 9205 human and 2484 *P. falciparum* genes.

We also leveraged the RNA-seq data to examine, within each infection, allelic variations at SNPs located in expressed parasite transcripts^15,17^ and determined that 87 of the 136 infections (64%) were polyclonal (Supplementary Data 1, see Materials and Methods for details).

### Variations in host and parasite gene expression are primarily driven by the child’s age and the infection’s parasitemia

To understand the contributions of different parameters to variations in host and parasite gene expression during symptomatic malaria episodes, we estimated the proportion of the variance in gene expression explained by the clinical and epidemiologic variables described in Table 1, considering all variables simultaneously^18^. Overall, most of the variance in host and parasite gene expression was caused by inter-individual differences in gene expression (labeled “residuals” in our model).

**Fig. 1:**
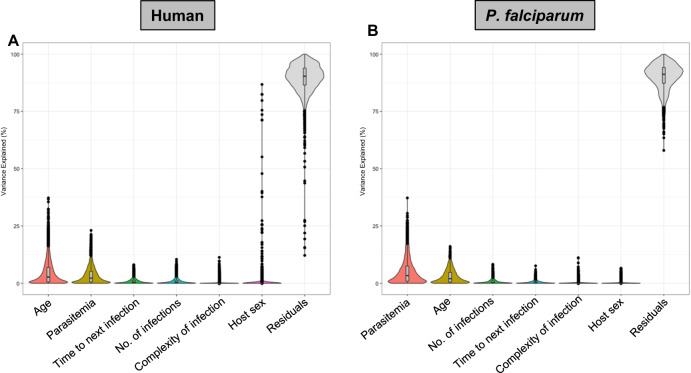
Percentage of the variance in host and parasite gene expression explained by each variable considered. Each violin plot shows the percentage of variance (y-axis) explained by each variable (x-axis) for each (**A**) human gene (*n* = 9205) and (**B**) *P. falciparum* gene (*n* = 2484 genes) based on the analyses of 136 infections. Each black dot represents one gene. The internal boxplot shows the median as a horizontal bar and the interquartile range of variance for each variable. “Residuals” indicates the percentage of the variance in gene expression not explained by any of the variables considered (i.e., driven by remaining inter-individual differences).

From the factors we examined, only two variables contributed substantially to the overall variance in host gene expression: the child’s age explained on average 5% of the overall variance in host gene expression (and between 0% and 39% of the variance of individual genes), while the infection’s parasitemia explained on average 3% (range = 0% to 23%) (Fig. 1A, Table 2). The remaining variables - the number of subsequent infections, the time to the next infection, the complexity of infection, or the sex of the participant—contributed very little to the overall variance in host gene expression (Fig. 1A, Table 2). Although sex differences in the clearance of *P. falciparum* infections have been described^19^, we did not observe significant sex differences in our data, with the exception of a small number of genes located on the sex chromosomes whose expression was significantly impacted by the sex of participants (Supplementary Data 2).

**Table 2:** Differential gene expression

Variable		Human DEGs^a^	%age Human gene expression explained	*P. falciparum* DEGs^a^	%age *P. falciparum* gene expression explained
Parasitemia (parasites/µL)	Unadjusted (Adjusted^b^)	3221 (21)	3.36%	1779 (71)	5.24%
Participant’s age (years)	Unadjusted (Adjusted^b^)	4174 (1485)	4.52%	833 (6)	3.02%
No. subsequent symptomatic episodes	-	13	0.93%	6	0.73%
Time to next symptomatic malaria episode	-	0	0.87%	0	0.52%
Complexity of infection	-	0	0.23%	0	0.26%
Participant’s sex	-	68	0.33%	1	0.21%

The table shows the number of human and *P. falciparum* genes associated with each clinical and epidemiologic variable (FDR = 0.1), before (unadjusted) and after (adjusted) adjusting for human immune cell or parasite developmental stage composition, respectively. All models included the host sex and month of infection as covariates, as well as age and parasitemia where appropriate. The mean proportion of overall variance in gene expression explained by each variable is also indicated for sake of comparison (see Fig. 1).

^a^DEGs = differentially expressed genes (FDR = 0.1).

^b^Adjusted for host immune cell or parasite developmental stage composition.

Similarly, the variance in *P. falciparum* gene expression was partially explained by the parasitemia of the infection (median = 3%, range = 0% to 37%) and the child’s age (median = 2%, range = 0–16%), with the remaining variables explaining very little of the gene expression variance (Fig. 1B, Table 2).

We then statistically tested which specific host and parasite genes were differentially expressed according to these variables, accounting for the child’s sex and the month of the infection, and correcting for multiple testing by false discovery rate.

Consistent with the results of the analysis of variance presented above, many host and parasite genes were significantly associated with the child’s age and the infection’s parasitemia, while the number of subsequent infections and time to the next infection, the complexity of infection, or the sex of the participant were associated with only a small number of genes (Table 2, Supplementary Data 3).

### Host gene expression associated with parasitemia is mainly driven by differences in the proportions of neutrophils and T cells

**Fig. 2:**
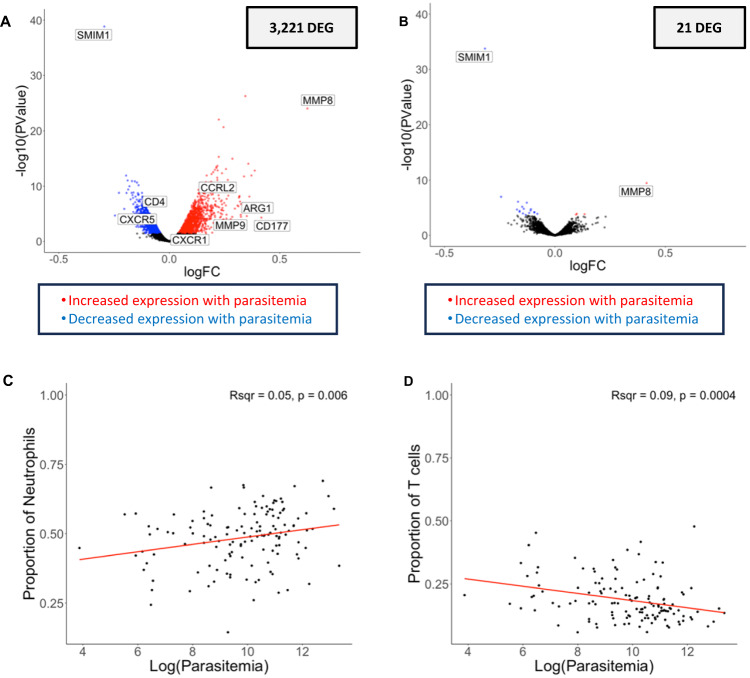
Host gene expression and parasitemia. The volcano plots show the association of host gene expression with the log of the parasitemia before (**A**) and after (**B**) adjusting for immune cell composition using a quasi-likelihood negative binomial generalized model. Each point represents one gene, displayed according to its *p*-value (y-axis) and log fold-change (x-axis). Blue and red points represent genes that were significantly more expressed in low and high parasitemia infections, respectively, corrected for multiple testing using false discovery rate (FDR) (FDR = 0.1). Correlation of the proportion of neutrophils (**C**) or T cells (**D**) (y-axis), estimated by gene expression deconvolution, with the log of the parasitemia (x-axis) using linear regression (respectively, Pearson’s *R*^2^ = 0.05, *p* = 0.006 and Pearson’s *R*^2^ = 0.09, *p* = 0.0004). *DEG = differentially expressed gene. Note that since we measured gene expression correlated with parasitemia as a continuous variable, the log fold-change reflects the change in expression of each gene with each unit of parasitemia, which can be smaller than typical log fold-change values that measure differences in expression between two groups. (*N* = 136 individuals).

3221 human genes were associated with the infection’s parasitemia, after accounting for the host’s age, sex, and the month of the infection (Table 2, Fig. 2A). Many of the genes whose expression was positively correlated with parasitemia were neutrophil surface markers (e.g., CD177^20^), granule/secretory vesicle proteins (e.g., MMP8^21^, MMP9^22^, ARG1^23^), and genes involved in neutrophil recruitment (e.g., CXCR1^24^, CCRL2^25^) (Supplementary Data 3). Conversely, the expression level of many genes related to T cells (e.g., CD3^26^, CD4, CD8^27^, and CXCR5^28^) were negatively associated with parasitemia (Supplementary Data 3).

To evaluate whether these differences in gene expression were indicative of differences in cell composition, we used gene expression deconvolution^10^ to estimate the proportion of different white blood cell subsets in each sample (Supplementary Data 4) and tested whether the relative proportions of those immune cell types were associated with parasitemia. Consistent with previous reports^29,30^, we found that the relative proportion of neutrophils was significantly associated with parasitemia (*R*^2^ = 0.05, *p* = 0.006), with high parasitemia infections displaying a greater proportion of neutrophils (Fig. 2C). Neutrophils are important first responders in the innate immune system^31,32^ and have been reported to interact with *Plasmodium*-infected RBCs through phagocytosis and NET formation^31–35^. Our results could indicate that neutrophils are released from the bone marrow into the peripheral blood proportionally to the number of parasites present, as circulating neutrophils attempt to combat the infection. Alternatively, these findings could indicate that high parasitemia infections are characteristic of children with less developed immunity, relying more on a strong innate response (note however that not all children with high parasitemia infections were young, Supplementary Fig. 2).

Conversely, we found that the relative proportion of T cells was negatively associated with parasitemia (*R*^2^ = 0.09, *p* = 0.004): low parasitemia infections had, proportionally, more T cells than high parasitemia infections (Fig. 2D). Several non-exclusive mechanisms could explain this finding: (i) T cell-mediated reduction of parasitemia (or initial reduction of hepatocyte infection, inhibiting blood stage development, which cannot be measured in this study), (ii) parasite-mediated T cell inhibition, (iii) lack of T cell stimulation at low parasitemia, and/or (iv) T cell extravasation into secondary lymphoid tissues (so that they are missed in our peripheral blood samples). Due to the relatively limited resolution of gene expression deconvolution that may hamper an accurate estimation of rarer cell types^36,37^, and to prevent data overfitting, we initially chose to conservatively estimate the proportion of only eight broadly-defined WBC subsets: neutrophils, T cells, B cells, mast cells, eosinophils, monocytes, NK cells and plasma cells. However, to assess whether a specific T cell subset was driving the correlation with parasitemia, we reiterated our gene expression deconvolution analysis and estimated the relative proportion of 22 immune cell subtypes included in our reference dataset, including seven different T cell populations^11^ (Supplementary Data 4). We found that the proportion of naïve CD4 T cells (*p* = 0.00012, *R*^2^ = 0.10) and regulatory T cells (Treg) (*p* = 1.55 × 10^−^^5^, *R*^2^ = 0.13) were negatively correlated with parasitemia, while activated memory CD4 T cells (*p* = 0.013, *R*^2^ = 0.038) were positively correlated with parasitemia (Supplementary Fig. 3). Some studies in mice have suggested that parasite-specific CD4 T cells directly reduce parasitemia^38^. Our observed higher abundance of naïve CD4 T cells in low parasitemia infections in human children could indicate that, at the time of the blood collection, the parasitemia had already been controlled by the abundance of CD4 T cells. Tregs have been shown in human studies to have a complex and exposure-dependent role during *P. falciparum* infection and their relationship with parasitemia remains controversial^39–41^. These cells expand after an immune response to modulate immunopathology induced by other cell types^39^. These findings suggest either (i) Treg expansion occurs after parasitemia has been controlled by other cell types (e.g., CD4 T cells) or (ii) high parasitemia infections do not efficiently induce a Treg response, which could contribute to further immunopathology from these infections. Our observed enrichment for activated memory CD4 T cells in high parasitemia infections likely reflects the expansion of *P. falciparum*-specific memory cells in order to combat the infection in children who have developed some immunity from prior infections.

The analyses described above rely on estimations of the relative proportions of WBC subsets and the observations of more neutrophils and fewer T cells in high parasitemia infections might therefore not be independent. To better interpret these results, it will therefore be important to follow up on these observations with techniques such as flow cytometry that provides absolute quantitation and greater resolution. In addition, while blood samples were collected at the time that each individual presented to clinic with symptoms of malaria, it is possible that samples of different parasitemia were collected at different times after infection. This limitation (inherent to human field studies) could contribute to our observed differences in neutrophil and T cell proportions. Future work in animal models, where the infection and sampling times can be tightly controlled, could help in clarifying the relationship between T cells and parasitemia (although important differences between animal models and human pathology exist^42^).

We then tested whether some of the differences in gene expression associated with parasitemia remained significant after accounting for the relative proportion of each major immune cell subset. After adjusting for differences in cell composition, only 21 of the initial 3,221 differentially expressed host genes remained significantly associated with parasitemia (Fig. 2B), suggesting that gene expression differences associated with parasitemia were mainly driven by heterogeneity in immune cell types, rather than differences in gene regulation. Two genes stood out in this analysis: SMIM1 and MMP8. SMIM1 is the surface marker for the Vel blood group^43^ and is an under studied red blood cell (RBC) surface marker that may regulate RBC formation and hemoglobin concentration^43^. SMIM1 has not been previously reported in malaria studies, but the correlation between its expression and parasitemia could indicate that it plays a role in regulating parasite development, possibly by modulating the amount of available hemoglobin (an important nutrient source for the parasites). Expression of MMP8, a neutrophil granule protein^21^, has been shown to be elevated in the serum of individuals with uncomplicated malaria^44^ and has been associated with malaria severity, particularly with cerebral malaria^8,45^. Although none of the individuals included in our study experienced cerebral malaria, it is possible that individuals with high parasitemia infections (and higher expression of MMP8) experienced more severe symptoms (symptom severity was not measured precisely in this cohort). Alternatively, this pattern of MMP8 expression could suggest a parasitemia-dependent neutrophil regulation, which warrants further study, particularly as it relates to immunopathology induced by this enzyme and its impact on disease severity.

Parasitemia and host age are not independent due to the gradual development of anti-malarial immunity with age and repeated exposures^46–48^ (Supplementary Fig. 2). Therefore, it is possible that, by adjusting the statistical analyses for age, we may have over-adjusted for genes that were correlated with both age and parasitemia. To attempt to address this confounding issue, we repeated our analyses, without adjusting for age but using the largest possible subset of children in one narrow age range: four- and five-year-old children (*N* = 47). After adjusting for immune cell composition, we identified 143 genes associated with parasitemia (Supplementary Fig. 4, Supplementary Data 5), including neutrophil effector proteins (e.g., MMP9^22^, LTF^22^, PGLYRP1^49^) and markers of neutrophil activation (e.g., CD177^20^, CD300H^50^) that displayed higher expression in high parasitemia infections. This result suggests that, in addition to the increase in circulating neutrophils, neutrophil regulation or proportions of different neutrophil subtypes (which were not sub-divided in our reference dataset) may also vary according to parasitemia, which should be investigated in more detail with higher resolution techniques such as flow cytometry. Similarly, we found that, in addition to a lower proportion of T cells, an important T cell growth factor (e.g., IL15^51^) was negatively associated with parasitemia, suggesting differences in T cell activation at different levels of parasitemia (Supplementary Data 5). This finding further supports the hypothesis of an inefficient stimulation of T cells overall at low parasitemia and/or rapid control of parasitemia by memory cells before the time of sampling and warrants further investigation by classic immunology methods and flow cytometry in future cohorts. Interestingly, we also detected multiple interferon (IFN) stimulated genes that were negatively associated with parasitemia, independently of age (Supplementary Data 5). While these genes can be expressed by a variety of cell types, this observation likely indicates that (i) efficient IFN signaling leads to a dramatic reduction in parasitemia and (ii) there is a critical threshold of parasites required to trigger efficient IFN signaling. These findings further highlight the complexity and heterogeneity of the anti-malarial immune response among children and warrant studies, with larger sample sizes, to fully disentangle its relationship with parasitemia, independently from the patient’s age.

### Parasite gene expression associated with parasitemia is primarily driven by differences in stage composition

**Fig. 3:**
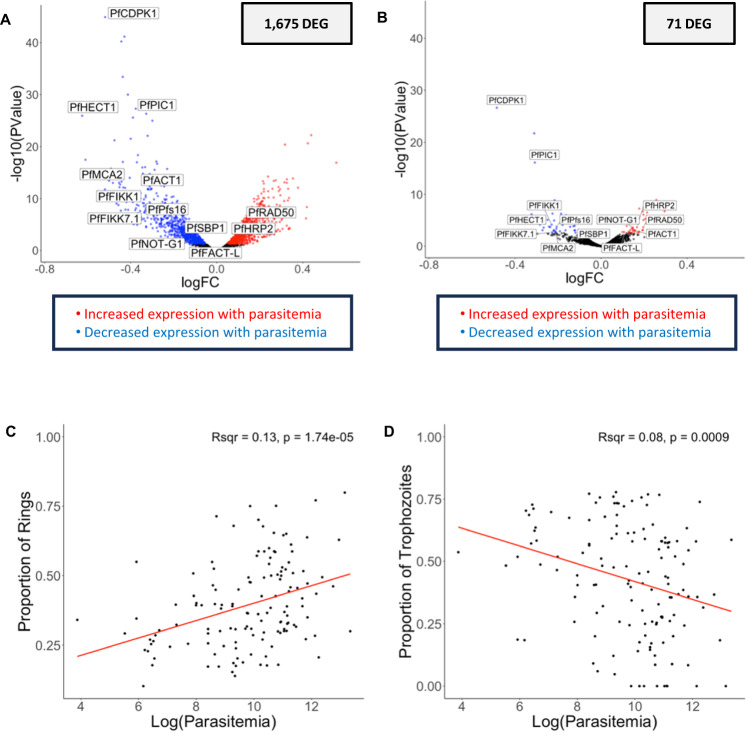
Parasite gene expression and parasitemia. The volcano plots show parasite gene expression associated with the parasitemia, unadjusted (**A**) and adjusted (**B**) for developmental stage composition using a quasi-likelihood negative binomial generalized model. Each point represents one gene, displayed according to its p-value (y-axis) and log fold-change (x-axis). Blue and red points represent genes that were significantly more expressed in low and high parasitemia infections, respectively, correcting for multiple testing using FDR (FDR = 0.1). Correlation of the proportion of rings (**C**) or trophozoites (**D**) (y-axis), estimated from gene expression deconvolution, with the log of the parasitemia (x-axis) using linear regression (respectively Pearson’s *R*^2^ = 0.13, *p* = 1.74 × 10^−5^ and Pearson’s *R*^2^ = 0.08, *p* = 0.0009). *DEG = differentially expressed gene. (*N* = 136 individuals).

We identified 1675 *P. falciparum* genes associated with parasitemia (Fig. 3A, Supplementary Data 6). To determine whether these differences in gene expression were due to differences in developmental stage composition among samples, we estimated the relative proportion of each developmental stage in each sample by gene expression deconvolution^13^ (Supplementary Data 4). The relative proportion of ring-stage parasites was positively correlated with parasitemia (*R*^2^ = 0.13, *p* = 1.74 × 10^−5^) (Fig. 3C), while the relative proportion of trophozoite stage parasites was negatively correlated with parasitemia (*R*^2^ = 0.08, *p* = 0.0009) (Fig. 3D). Mature asexual *P. falciparum* parasites typically sequester in the tissues of infected patients and ring-stage parasites largely predominate in the peripheral blood^52–54^. However, since ring-stage *Plasmodium* parasites are less transcriptionally active than other developmental stages^55^, gene expression data can overestimate the relative proportion of mature stages (but proportionally in all samples^13^). The observed differences in developmental stage composition associated with parasitemia could therefore suggest that (i) mature parasite sequestration is more efficient at high parasitemia and/or (ii) that the regulation of intraerythrocytic development is parasitemia-dependent. Consistent with the latter hypothesis, several genes involved in asexual development remained negatively associated with parasitemia after adjusting for cell composition (e.g., PfCDPK1^56,57^, PfPIC1^58^, PfHECT1^59^, see below for details) (Fig. 3B), possibly indicating that, when parasitemia is high, parasites downregulate key genes to slow down their growth rate. Mouse models of *P. berghei* have recently shown that systemic host inflammation can slow maturation of the asexual parasites, suggesting that this parasitemia-dependent growth regulation is host mediated^60^. Our results support this model in humans with uncomplicated disease and are consistent with results from human studies of severe^61^ and asymptomatic^62,63^ disease states: higher parasitemia infections lead to more inflammation (see human gene expression results above) and this inflammatory environment could possibly explain the differences in asexual stage regulation observed in the *P. falciparum* gene expression.

We then tested whether the expression levels of some *P. falciparum* genes were associated with parasitemia after accounting for differences in developmental stage composition. After adjusting for stage composition, only 71 genes remained significantly associated with parasitemia (Fig. 3B) including known antigens (e.g., PfHRP2^64^), genes involved in DNA replication (e.g., PfRAD50^65^) and parasite asexual replication (e.g., PfACT1^66^), which were positively associated with parasitemia, and genes involved in asexual development (e.g., PfCDPK1^56,57^, PfPIC1^58^, PfHECT1^59^) and erythrocyte surface remodeling (e.g., PfFIKK1^67,68^, PfFIKK7.1^67,68^, PfSBP1^69^), which were negatively associated with parasitemia (Supplementary Data 6).

As with the human gene expression analysis, since parasitemia and age are correlated, we may have missed parasite genes associated with parasitemia by over-adjusting our model. We therefore repeated our gene expression analyses with the subset of 47 four- to five-year-old children, described above, and identified 421 genes associated with parasitemia after adjusting for developmental stage composition (Supplementary Fig. 5, Supplementary Data 5). Consistent with our findings from the full cohort, we found several genes involved in invasion (e.g., PfGBP130^70^, PfPK2^71^, PfTrx-mero^72^) and replication (e.g., genes involved in cell cycle progression and chromosome organization) to be negatively associated with parasitemia. Interestingly, several of these additional genes associated with parasitemia are consistent with parasitemia-dependent host-pathogen interactions. The expression of PfHMGB1, a gene that promotes host TNFα secretion in mouse models^73^, was negatively correlated with parasitemia, suggesting that parasites modulate the host inflammatory response to increase their survival. In addition, PfEH1 and PfEH2^74^ were also less expressed in high parasitemia infections. These enzymes degrade erythrocyte-derived lipid signaling molecules^74^ thereby reducing endothelial activation and decreasing the expression of ICAM1, an important ligand for parasite sequestration^53^. At high parasitemia, lower expression of these enzymes may maintain a high level of ICAM1 expression in the endothelium, allowing for more efficient sequestration of parasites within tissues (consistent with our stage composition analyses) and facilitating evasion of host immunity. These possible mechanisms of parasitemia-dependent modulation of host inflammation and parasite sequestration will need to be validated but provide exciting hypotheses for studying unexplored density-dependent host-pathogen interactions in malaria infections.

### Host gene expression associated with participant age is partially explained by differences in immune cell composition

**Fig. 4:**
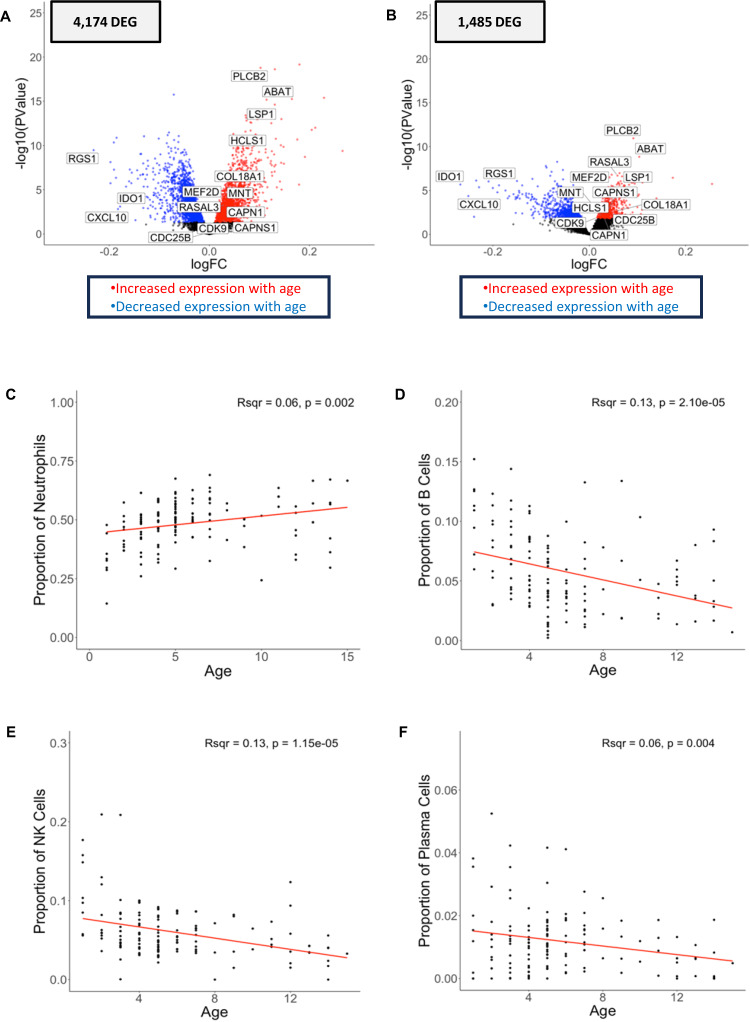
Host gene expression and child’s age. The volcano plots show the association between each human gene’s expression and the child’s age at the time of the infection, before (**A**) and after (**B**) adjusting for differences in immune cell composition using a quasi-likelihood negative binomial generalized model. Each dot represents one gene and is displayed according to its log10 *p*-value (y-axis) and fold-change (x-axis). Blue and red points represent differentially expressed genes that were more expressed in younger and older children, respectively, corrected for multiple testing using FDR (FDR = 0.1). Correlation of the proportion of neutrophils (**C**), B cells (**D**), NK cells (**E**), and Plasma cells (**F**) (y-axis), estimated from gene expression deconvolution, with the age of the child in years (x-axis) using linear regression (respectively, Pearson’s *R*^2^ = 0.06, *p* = 0.002; Pearson’s *R*^2^ = 0.13, *p* = 2.10 × 10^−5^; Pearson’s *R*^2^ = 0.13, *p* = 1.15 × 10^−5^; and Pearson’s *R*^2^ = 0.06, *p* = 0.004). Note that different ranges for the y-axis in (**C**, **D**, **E**, **F**) due to differences in cell proportions. *DEG = differentially expressed gene. (*N* = 136 individuals).

We identified 4174 genes with expression significantly associated with host age (which ranged from 1 to 15 years old in our cohort) (Table 2, Fig. 4A, Supplementary Data 3). To determine whether these differences in gene expression were explained by differences in cell composition among samples, we examined the correlation between host age (as a continuous variable) and the relative proportion of each immune cell type. The proportion of neutrophils was positively associated (*R*^2^ = 0.07, *p* = 0.0019), and the proportions of B cells (*R*^2^ = 0.13, *p* = 2.10 × 10^−5^), NK cells (*R*^2^ = 0.13, *p* = 1.15 × 10^−5^) and plasma cells (*R*^2^ = 0.06, *p* = 0.004) were negatively associated with age (Fig. 4C–F).

However, in contrast to the gene expression differences associated with parasitemia, host gene expression associated with age was only partially explained by changes in cell composition: over one third of the differentially expressed genes (N = 1485) remained significantly associated with age after adjusting for WBC composition (Table 2, Fig. 4B), and none of these remaining differentially expressed genes overlapped with those associated with parasitemia. To contextualize the genes that remained associated with age after adjusting for cell composition, we used the KEGG database^75–77^ to examine their distribution among key relevant pathways using Pathview^78,79^. The gene expression patterns in older children were consistent with activation of adaptive immunity, including activation of platelets (e.g., PLCB2, Pi3K) (Supplementary Fig. 6A), T cell metabolism (e.g., ABAT^80^, MEF2D^81,82^), and neutrophil inflammatory response (e.g., RASAL3^83^, LSP1^84–86^) (note that neutrophil activation is not represented in the KEGG database) (Supplementary Data 3). We further found increased expression of several genes involved in TCR (Supplementary Fig. 6B) and BCR (Supplementary Fig. 6C) signaling pathways. While acquisition of immune memory to *P. falciparum* is complex and not entirely understood^87–89^, taken together, these patterns are consistent with a T cell memory response. Neutrophil and platelet activation can enhance the adaptive immune response in general^90–92^, as well as memory CD4 T cell responses, specifically^90,93,94^. Memory CD4 T cell responses have also been observed upon repeated infections in mouse models^95–97^. After re-infection, *Plasmodium*-specific memory CD4 T cells rapidly proliferate to respond to the pathogen. Indeed, we also observed increased expression of several genes involved in cell proliferation^98^ (e.g., CAPN1, CAPNS1, CDC25B, CDK9, COL18A1, HCLS1, MNT) positively correlated with age (Supplementary Data 3).

Interestingly, several genes involved in the regulation of the actin cytoskeleton (Supplementary Fig. 6D) and focal adhesion (Supplementary Fig. 6E) pathways were also positively correlated with age, which could indicate immune synapse formation and/or leukocyte extravasation. Though not specific to memory lymphocytes, the immune synapse is required for activation of T and B cells^99^. The actin cytoskeleton has also been shown to undergo remodeling after successful TCR^100^ and BCR^101^ signaling. Because activation of memory lymphocytes is faster than naïve lymphocytes^102^, we speculate that the association of an activated adaptive response in older children at symptom presentation is consistent with a memory response after years of exposure and immune system aging.

The gene expression pattern in younger children was broadly suggestive of an innate inflammatory response, including genes whose expression is induced by interferon signaling (e.g., RGS1^103^, IDO1^104^, CXCL10^105^), NOD-like receptor (NLR) signaling (Supplementary Fig. 7A), Toll-like receptor (TLR) signaling (Supplementary Fig. 7B), phagocytosis (Supplementary Fig. 7C) and antigen presentation (Supplementary Fig. 7D). This innate-dominated immune environment in younger children (here, the lower limit of age being 1 year old with most children in the cohort being between 1 and 5 years old) is consistent with the clinical observation that immunological memory to malaria does not develop until later in adolescence and adulthood^89^. These findings also likely reflect the maturation of the immune system over time, which has been shown to impact anti-malarial immunity^47,48^, but has been largely under studied in healthy children in this age range, particularly in Sub-Saharan African populations.

Both TLR and NLR signaling are key components of the innate response to *Plasmodium* infection^106^. While it is still unclear how NLR signaling pathways impact anti-malarial immunity, hemozoin, a toxic byproduct of *Plasmodium* digestion of hemoglobin, can stimulate NLRP3 (an NLR) in vitro^107^. TLR recognition of *Plasmodium* has been better characterized^108^: TLR9 recognizes DNA-hemozoin complexes, TLR1/2 heterodimers recognize GPI anchors of *Plasmodium* proteins, and TLR7 and TLR8 recognize *Plasmodium* RNA. TLR and NLR signaling share a common result: the production of interferons, mediating host defense against the pathogen, as well as immunopathology^108^.

Because phagocytosis is a key component of antigen presentation^109^, these pathways are likely linked. Antigen presentation is a key step in bridging the innate and adaptive immune systems^109^ and our findings suggest that the innate immune system in younger children is actively responding to *P. falciparum* at the time of sample collection (i.e., at symptom presentation).

Overall, our findings highlight that different immune pathways are preferentially activated upon *P. falciparum* infection depending on the age of the child, progressing from a greater reliance on innate immunity to acquired immunity as a child ages. These findings provide a potential mechanism underlying the gradual acquisition of immunity, first against severe disease and eventually from all malaria symptoms as children age, as has been reported in several epidemiological^110,111^ and clinical studies^112^. While age in this context is likely to be at least partially a proxy for exposure to *P. falciparum*, as children in our cohort experience, on average, two malaria infections per transmission season^16^, both age and repeated exposure have been independently linked to clinical protection from malaria and development of anti-*Plasmodium* immunity in Ugandan children^46,113^. Additionally, studies of Indonesian adults have shown an age-dependent reduction in risk of malaria disease, independent of prior exposures, suggesting that development of the immune system with age influences clinical protection^47,48^. Here, our results provide evidence for age-dependent gene expression in Malian children, which could be linked to the development of clinical protection from malaria and will be important to validate with basic immunology methods and larger cohorts in future studies. Additionally, future studies with access to prior infection history to precisely account for malaria exposure in each child, independent of age, will be important in disentangling gene expression separately associated with age and exposure history.

Again, because age and parasitemia are correlated, we may miss genes that were truly associated with age by adjusting for parasitemia. We attempted to analyze subsets of children with similar parasitemia but of different ages by creating bins of parasitemia with a one log range, but the low sample sizes within each bin precluded meaningful analyses and further studies are necessary.

### The proportion of male gametocytes is associated with participant age

**Fig. 5:**
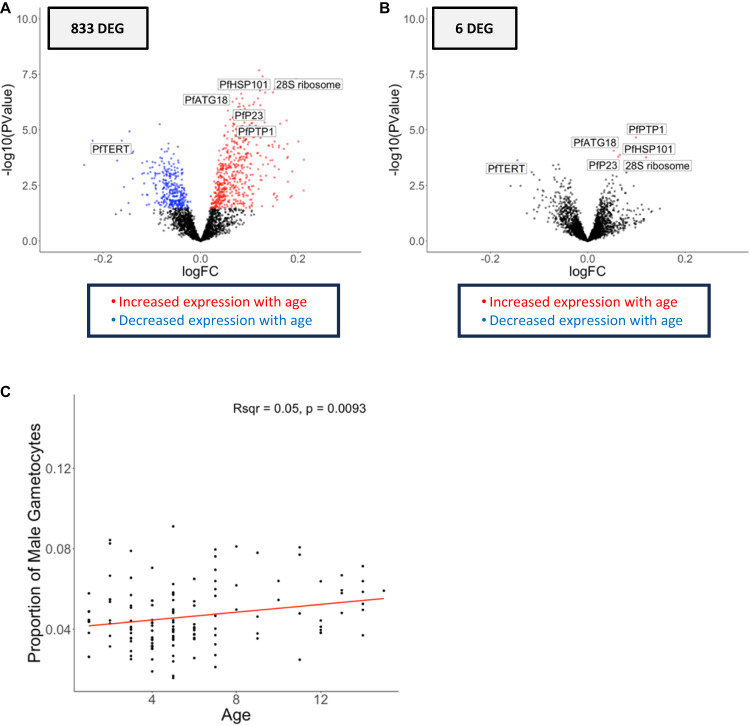
Parasite gene expression and child’s age. The volcano plots show parasite gene expression associated with the child’s age at the time of infection, before (**A**) and after (**B**) adjusting for developmental stage composition using a quasi-likelihood negative binomial generalized model. Each point represents one gene, displayed according to its *p*-value (y-axis) and fold-change (x-axis). Blue and red points represent differentially expressed genes that were more expressed in younger and older children, respectively, corrected for multiple testing using FDR (FDR = 0.1). Correlation of the proportion of male gametocytes (y-axis) inferred from gene expression deconvolution with the participant’s age in years (x-axis) using linear regression (Pearson’s *R*^2^ = 0.05, *p* = 0.0093) (**C**). Note that different ranges for the y-axis in C due to differences in cell proportions. *DEG = differentially expressed gene. (*N* = 136 individuals).

Out of 2,484 *Plasmodium* genes, 833 were associated with participant age (Table 2, Fig. 5A, Supplementary Data 6). Interestingly, the proportion of male gametocytes, determined by gene expression deconvolution, was positively associated with participant age (*R*^2^ = 0.049, *p* = 0.0093) (Fig. 5C). While gametocyte development is not completely understood, *Plasmodium* parasites have been shown to vary their sex ratio according to environmental changes^114,115^, including host immune status^114,116^. Consistent with our findings, a few studies have also reported a male-skewed gametocyte sex ratio in older *P. falciparum*-infected children^117,118^. Since variation in gametocyte sex ratio is thought to impact transmission success^115,119^ and male-dominated ratios have been shown to increase transmission from infected humans to mosquitos^119^, our observations could indicate that older children are more likely to contribute to disease transmission, consistent with previous epidemiology work from other settings with continuous, rather than seasonal transmission^120,121^. This result further highlights that malaria elimination initiatives should consider age (in addition to immunity status) when prioritizing interventions.

It is important to note here that participants were enrolled when they presented with (self-reported) malaria symptoms. It is possible that older children became symptomatic later in the course of one infection than younger children, due to increasing immunity with age^46,122^ and “older” infections are more likely to harbor higher gametocytemia, as *P. falciparum* gametocytes appear in the blood after about 2 weeks^114^. However, while age of infection may contribute to gametocytemia overall, it is unlikely that this entirely explains the male-skewed gametocytemia we observed here.

After accounting for differences in stage composition, only six *P. falciparum* genes remained associated with participant age (Table 2, Fig. 5B, Supplementary Data 6) and none of these six genes overlapped with those identified to be associated with parasitemia.

### Genes related to a type 1 IFN response are associated with greater numbers of subsequent malaria episodes

While the number of subsequent symptomatic malaria episodes in the study period was not a major driver of the host gene expression, 13 genes were significantly associated this variable. Three genes that are characteristic of (although not exclusive to) a type 1 IFN (IFN1) response, CXCL10^105^, SOCS1^123^, PLAAT4^124^, were negatively associated with the number of subsequent infections a child experienced (i.e., higher expression of these genes during one infection was associated with experiencing fewer subsequent infections) (Supplementary Fig. 8, Supplementary Data 3). The effects of IFN1 in response to malaria are variable and depend on both host and parasite genotype^108^, but they have been shown to influence T cell activation^125^ and antibody production^126^. CXCL10 expression can also lead to growth acceleration of *P. falciparum* in vitro^127^. Likewise, we found a positive correlation between CXCL10 with parasitemia, potentially suggesting a positive feedback-like interaction between the host and parasite, whereby parasites stimulate an IFN1 response in the host, leading to CXCL10 production, which can both increase parasitemia and modulate protection against future infections by influencing the adaptive immune response. Future mechanistic immunology studies are necessary to precisely disentangle this relationship.

### Gametocyte markers are associated with susceptibility to future malaria episodes

Similarly to the host gene expression, few parasite genes were significantly associated with the number of subsequent symptomatic infections in the study period (N = 6) but those included known regulators of gametocytogenesis (PfG27/25 and PfAP2-G) (Supplementary Fig. 9, Supplementary Data 6). This observation is interesting given previous reports linking higher gametocyte density with an anti-inflammatory environment^128,129^. This could suggest that failure to develop a sufficient inflammatory response to one infection could promote gametocyte production and reduce the development of long-term, protective immunity. Again, because gametocytes are the transmissible stage of *P. falciparum*, it is important to understand the interplay between susceptibility and gametocytogenesis to both protect susceptible children and prevent transmission of the parasites.

## Methods

### Ethics approval and consent

Individual informed consent/assent was collected from all children and their parents. The study protocol and consent/assent processes were approved by the institutional review boards of the Faculty of Medicine, Pharmacy and Dentistry of the University of Maryland, Baltimore, and of the University of Sciences, Techniques and Technologies of Bamako, Mali (IRB numbers HCR-HP-00041382 and HP-00085882).

### Samples

We selected 136 whole blood samples, collected directly in PAXgene blood RNA tubes, from children experiencing a symptomatic uncomplicated malaria episode caused by *Plasmodium falciparum* parasites. The majority of samples were collected during the peak of malaria transmission in Mali (~June–December). The numbers of symptomatic cases occurring each month, based on the entire 4-year cohort, are depicted in Supplementary Fig. 10. The presence of parasites and the parasite species were initially determined by light microscopy using thick blood smears. Sex and gender were not explicitly considered in the study design, but samples were selected in approximately equal proportion from male and female children. All infections were successfully treated with anti-malarial drugs according to the Mali National Malaria Control Program standards.

### Case definition

Children were classified, by the field clinicians, as experiencing symptomatic uncomplicated malaria if they (i) sought treatment from the study clinic, (ii) experienced symptoms consistent with malaria (i.e., fever, headache, joint pain, abdominal pain, vomiting or diarrhea), (iii) *Plasmodium falciparum* parasites were detected, at any density, by thick blood smear, and (iv) if they lacked any signs of severe malaria (e.g., coma, seizures, severe anemia)^16^.

Weighted number of and time between subsequent infections: To account for variations in risk of transmission throughout the study period, we weighted the number of, and time between, infections for the relative risk of malaria during each child’s person-time in the study. To calculate the number of subsequent infections, we first calculated, for the whole cohort, the number of malaria cases per month divided by the total number of children followed-up in that month (month-weight). Next, we calculated each child’s person-time left in the study after our sequenced infection by taking the sum of the month-weights for each month during which a child was enrolled after the date of our sequenced infection. Finally, we divided each child’s number of symptomatic malaria episodes occurring after our sequenced infection by their person-time remaining. To calculate the time to the next subsequent symptomatic malaria episode for each child, we summed the month-weights for each month between our sequenced infection and the next documented symptomatic malaria episode.

### Generation of RNA-seq data

We extracted RNA from whole blood using MagMax blood RNA kits (Themo Fisher). Total RNA was subjected to rRNA depletion and polyA selection (NEB) before preparation of stranded libraries using the NEBNext Ultra II Directional RNA Library Prep Kit (NEB). cDNA libraries were sequenced on an Illumina NovaSeq 6000 to generate ~55-130 million paired-end reads of 75 bp per sample. To confirm that *P. falciparum* was responsible for each malaria episode, we first aligned all reads from each sample using hisat2 v2.1.0^130^ to a fasta file containing the genomes of all *Plasmodium* species endemic in Mali downloaded from PlasmoDB^131^ v55: *P. falciparum* 3D7*, P. vivax* PvP01*, P. malariae* UG01, and *P. ovale curtisi* GH01. After ruling out co-infections and misidentification of parasites, we aligned all reads using hisat2 to a fasta file containing the *P. falciparum* 3D7 and human hg38 genomes (i) using default parameters and (ii) using (--max-intronlen 5000). Reads mapping uniquely to the hg38 genome were selected from the BAM files generated with the default parameters. Reads mapping uniquely to the *P. falciparum* genome were selected from the BAM files generated with a maximum intron length of 5000 bp. PCR duplicates were removed from all files using custom scripts. We then calculated read counts per gene using gene annotations downloaded from PlasmoDB (*P. falciparum* genes) and NCBI (human genes) and the subread featureCounts v1.6.4^132^.

### Gene expression analysis

Read counts per gene were normalized into counts per million (CPM), separately for human and *P. falciparum* genes. To filter out lowly expressed genes, only human or *P. falciparum* genes that were expressed at least at 10 CPM in >50% of the samples were retained for further analyses (9205 and 2484 genes, respectively). Read counts were normalized via TMM for differential expression analyses. Statistical assessment of differential expression was conducted, separately for the human and *P. falciparum* genes, in edgeR (v 3.32.1)^133^ using a quasi-likelihood negative binomial generalized model (i) with and without adjusting for proportion of the major human immune cell types for human genes and (ii) with and without adjusting for proportion of each parasite developmental stage for *Plasmodium* genes. Age and parasitemia were considered as continuous variables using edgeR^133^. We log-transformed parasitemia estimates to fit the data to a normal distribution. Models used to estimate the gene expression associated with parasitemia also included host age, month of infection, and host sex as covariates. Models used to estimate the gene expression associated with host age also included parasitemia, month of infection, and host sex as covariates. We also explicitly tested whether gene expression was associated with host sex and reported these results in the main text. All results were corrected for multiple testing using FDR^134^.

### Gene expression deconvolution

CIBERSORTx^10^ was used to estimate, in each sample, the proportion of (i) human immune cell types and (ii) *Plasmodium* developmental stages, separately, directly from the RNA-seq data. To deconvolute human gene expression profiles, we used as a reference LM22^11^, a validated leukocyte gene signature matrix which uses 547 genes to differentiate 22 immune subtypes (collapsed to eight categories in our analysis to prevent data sparsity). A custom signature matrix derived from *P. berghei* scRNA-seq data^135^ was used for *P. falciparum* stage deconvolution, using orthologous genes between the two species^13^ which is available at https://github.com/tebbenk/GED.

### Complexity of infection

We used GATK GenotypeGCVFs^136^ to call variants in all samples directly from the RNA-seq reads and analyze the complexity of each infection examined. While this pipeline was initially developed for analyzing whole genome sequence data, we previously showed that it can be applied to RNA-seq data^15^. Briefly, we filtered the genotype file to retain only positions that had a maximum of two alleles, no more than 20% missing information and to remove positions within *Plasmodium* multi-gene families (due to inaccurate mapping of reads within these regions because of high sequence variability)^15^. To determine the complexity of each infection (i.e., monoclonal vs. polyclonal), we then estimated *F*_ws_ from the filtered genotype file from GATK using moimix^137^. Samples with *F*_ws_ > 0.95 were considered monoclonal and *F*_ws_ < 0.95 polyclonal.

### Reporting summary

Further information on research design is available in the Nature Portfolio Reporting Summary linked to this article.

### Supplementary information

Supplementary Information

Peer Review File

Description of Additional Supplementary Files

Supplementary Data 1

Supplementary Data 2

Supplementary Data 3

Supplementary Data 4

Supplementary Data 5

Supplementary Data 6

Reporting Summary

## Data Availability

The sequence data generated in this study have been deposited in the NCBI Sequence Read Archive under the BioProject PRJNA962942. All relevant metadata are provided in Supplementary Data 1. Supplementary Data 1 through 6 are also available at https://github.com/tebbenk/symptomatic_malaria.

## Code availability

Custom scripts are available on Github at https://github.com/tebbenk/symptomatic_malaria and 10.5281/zenodo.10631324^138^.

## References

[1] World malaria report 2022. Geneva: World Health Organization; 2022. Licence: CC BY-NC-SA 3.0 IGO.

[2] Phillips MA (2017). Malaria. Nat. Rev. Dis. Prim..

[3] Milner DA (2018). Malaria pathogenesis. Cold Spring Harb. Perspect. Med.

[4] Tran TM (2016). Transcriptomic evidence for modulation of host inflammatory responses during febrile Plasmodium falciparum malaria. Sci. Rep..

[5] Portugal, S. et al. Malaria-associated atypical memory B cells exhibit markedly reduced B cell receptor signaling and effector function. *Elife* **4**. 10.7554/eLife.07218 (2015) 10.7554/eLife.07218 PMC4444601 25955968

[6] Chou ES (2018). A high parasite density environment induces transcriptional changes and cell death in Plasmodium falciparum blood stages. FEBS J..

[7] Warimwe GM (2013). Plasmodium falciparum var gene expression homogeneity as a marker of the host-parasite relationship under different levels of naturally acquired immunity to malaria. PLoS One.

[8] Lee, H. J. et al. Integrated pathogen load and dual transcriptome analysis of systemic host-pathogen interactions in severe malaria. *Sci. Transl. Med.* **10**. 10.1126/scitranslmed.aar3619 (2018) 10.1126/scitranslmed.aar3619 PMC6326353 29950443

[9] Yamagishi J (2014). Interactive transcriptome analysis of malaria patients and infecting Plasmodium falciparum. Genome Res..

[10] Newman AM (2019). Determining cell type abundance and expression from bulk tissues with digital cytometry. Nat. Biotechnol..

[11] Chen B, Khodadoust MS, Liu CL, Newman AM, Alizadeh AA (2018). Profiling tumor infiltrating immune cells with CIBERSORT. Methods Mol. Biol..

[12] Newman AM (2015). Robust enumeration of cell subsets from tissue expression profiles. Nat. Methods.

[13] Tebben K, Dia A, Serre D (2022). Determination of the stage composition of plasmodium infections from bulk gene expression data. mSystems.

[14] Kim A, Popovici J, Menard D, Serre D (2019). Plasmodium vivax transcriptomes reveal stage-specific chloroquine response and differential regulation of male and female gametocytes. Nat. Commun..

[15] Bradwell KR (2020). Host and parasite transcriptomic changes upon successive Plasmodium falciparum Infections in early childhood. mSystems.

[16] Coulibaly D (2014). Stable malaria incidence despite scaling up control strategies in a malaria vaccine-testing site in Mali. Malar. J..

[17] Tebben K (2023). Malian children infected with Plasmodium ovale and Plasmodium falciparum display very similar gene expression profiles. PLoS Negl. Trop. Dis..

[18] Hoffman GE, Schadt EE (2016). variancePartition: interpreting drivers of variation in complex gene expression studies. BMC Bioinform..

[19] Briggs, J. et al. Sex-based differences in clearance of chronic Plasmodium falciparum infection. *Elife* **9**. 10.7554/eLife.59872 (2020) 10.7554/eLife.59872 PMC7591246 33107430

[20] Hu N (2014). Differential expression of granulopoiesis related genes in neutrophil subsets distinguished by membrane expression of CD177. PLoS One.

[21] Owen CA, Hu Z, Lopez-Otin C, Shapiro SD (2004). Membrane-bound matrix metalloproteinase-8 on activated polymorphonuclear cells is a potent, tissue inhibitor of metalloproteinase-resistant collagenase and serpinase. J. Immunol..

[22] Borregaard N, Cowland JB (1997). Granules of the human neutrophilic polymorphonuclear leukocyte. Blood.

[23] Munder M (2005). Arginase I is constitutively expressed in human granulocytes and participates in fungicidal activity. Blood.

[24] Capucetti A, Albano F, Bonecchi R (2020). Multiple roles for chemokines in neutrophil biology. Front. Immunol..

[25] Del Prete A (2017). The atypical receptor CCRL2 is required for CXCR2-dependent neutrophil recruitment and tissue damage. Blood.

[26] Georgopoulos K, Galson D, Terhorst C (1990). EMBO J..

[27] Germain RN (2002). T-cell development and the CD4-CD8 lineage decision. Nat. Rev. Immunol..

[28] Hardtke S, Ohl L, Forster R (2005). Balanced expression of CXCR5 and CCR7 on follicular T helper cells determines their transient positioning to lymph node follicles and is essential for efficient B-cell help. Blood.

[29] Kotepui M (2015). Effects of malaria parasite density on blood cell parameters. PLoS One.

[30] Otterdal K (2018). Soluble markers of neutrophil, T-cell and monocyte activation are associated with disease severity and parasitemia in falciparum malaria. BMC Infect. Dis..

[31] Pollenus E, Gouwy M, Van den Steen PE (2022). Neutrophils in malaria: the good, the bad or the ugly?. Parasite Immunol..

[32] Babatunde KA, Adenuga OF (2022). Neutrophils in malaria: a double-edged sword role. Front. Immunol..

[33] Zelter T (2022). Neutrophils impose strong immune pressure against PfEMP1 variants implicated in cerebral malaria. EMBO Rep..

[34] Kho S (2019). Circulating neutrophil extracellular traps and neutrophil activation are increased in proportion to disease severity in human malaria. J. Infect. Dis..

[35] Aitken EH, Alemu A, Rogerson SJ (2018). Neutrophils and malaria. Front. Immunol..

[36] Tsoucas D (2019). Accurate estimation of cell-type composition from gene expression data. Nat. Commun..

[37] Jin H, Liu Z (2021). A benchmark for RNA-seq deconvolution analysis under dynamic testing environments. Genome Biol..

[38] Hirunpetcharat C, Finkelman F, Clark IA, Good MF (1999). Malaria parasite-specific Th1-like T cells simultaneously reduce parasitemia and promote disease. Parasite Immunol..

[39] Kurup SP (2017). Regulatory T cells impede acute and long-term immunity to blood-stage malaria through CTLA-4. Nat. Med..

[40] Walther M (2009). Distinct roles for FOXP3 and FOXP3 CD4 T cells in regulating cellular immunity to uncomplicated and severe Plasmodium falciparum malaria. PLoS Pathog..

[41] Boyle MJ (2015). Decline of FoxP3+ regulatory CD4 T cells in peripheral blood of children heavily exposed to malaria. PLoS Pathog..

[42] Georgiadou, A. et al. Comparative transcriptomic analysis reveals translationally relevant processes in mouse models of malaria. *Elife* **11**. 10.7554/eLife.70763 (2022) 10.7554/eLife.70763 PMC8747512 35006075

[43] Cvejic A (2013). SMIM1 underlies the Vel blood group and influences red blood cell traits. Nat. Genet..

[44] Dietmann A (2008). Matrix metalloproteinases and their tissue inhibitors (TIMPs) in Plasmodium falciparum malaria: serum levels of TIMP-1 are associated with disease severity. J. Infect. Dis..

[45] Georgiadou A (2021). Localised release of matrix metallopeptidase 8 in fatal cerebral malaria. Clin. Transl. Immunol..

[46] Rodriguez-Barraquer, I. et al. Quantification of anti-parasite and anti-disease immunity to malaria as a function of age and exposure. *Elife* **7**. 10.7554/eLife.35832 (2018) 10.7554/eLife.35832 PMC6103767 30044224

[47] Baird JK (1997). Age-dependent characteristics of protection v. susceptibility to Plasmodium falciparum. Ann. Tropical Med. Parasitol..

[48] Baird JK (1995). Host age as a determinant of naturally acquired immunity to Plasmodium falciparum. Parasitol. Today.

[49] Read CB (2015). Cutting Edge: identification of neutrophil PGLYRP1 as a ligand for TREM-1. J. Immunol..

[50] Niizuma K, Tahara-Hanaoka S, Noguchi E, Shibuya A (2015). Identification and characterization of CD300H, a new member of the human CD300 immunoreceptor family. J. Biol. Chem..

[51] Giri JG (1995). IL-15, a novel T cell growth factor that shares activities and receptor components with IL-2. J. Leukoc. Biol..

[52] David PH, Hommel M, Miller LH, Udeinya IJ, Oligino LD (1983). Parasite sequestration in Plasmodium falciparum malaria: spleen and antibody modulation of cytoadherence of infected erythrocytes. Proc. Natl Acad. Sci. USA.

[53] Berendt AR, Ferguson DJ, Newbold CI (1990). Sequestration in Plasmodium falciparum malaria: sticky cells and sticky problems. Parasitol. Today.

[54] Beeson JG, Brown GV (2002). Pathogenesis of Plasmodium falciparum malaria: the roles of parasite adhesion and antigenic variation. Cell Mol. Life Sci..

[55] Bozdech Z (2003). The transcriptome of the intraerythrocytic developmental cycle of Plasmodium falciparum. PLoS Biol..

[56] Kumar S (2017). PfCDPK1 mediated signaling in erythrocytic stages of Plasmodium falciparum. Nat. Commun..

[57] Bansal A (2013). Characterization of Plasmodium falciparum calcium-dependent protein kinase 1 (PfCDPK1) and its role in microneme secretion during erythrocyte invasion. J. Biol. Chem..

[58] Wichers JS (2021). Identification of novel inner membrane complex and apical annuli proteins of the malaria parasite Plasmodium falciparum. Cell Microbiol.

[59] Paul AS (2020). Co-option of Plasmodium falciparum PP1 for egress from host erythrocytes. Nat. Commun..

[60] Lansink, L. I. M. et al. Systemic host inflammation induces stage-specific transcriptomic modification and slower maturation in malaria parasites. *mBio*, e0112923. 10.1128/mbio.01129-23 (2023) 10.1128/mbio.01129-23 PMC10470790 37449844

[61] Tonkin-Hill GQ (2018). The Plasmodium falciparum transcriptome in severe malaria reveals altered expression of genes involved in important processes including surface antigen-encoding var genes. PLoS Biol..

[62] Andrade CM (2020). Increased circulation time of Plasmodium falciparum underlies persistent asymptomatic infection in the dry season. Nat. Med.

[63] Thomson-Luque R (2021). Plasmodium falciparum transcription in different clinical presentations of malaria associates with circulation time of infected erythrocytes. Nat. Commun..

[64] Poti KE, Sullivan DJ, Dondorp AM, Woodrow CJ (2020). HRP2: transforming malaria diagnosis, but with caveats. Trends Parasitol..

[65] Lee AH, Symington LS, Fidock DA (2014). DNA repair mechanisms and their biological roles in the malaria parasite Plasmodium falciparum. Microbiol Mol. Biol. Rev..

[66] Das S, Lemgruber L, Tay CL, Baum J, Meissner M (2017). Multiple essential functions of Plasmodium falciparum actin-1 during malaria blood-stage development. BMC Biol..

[67] Nunes MC, Goldring JP, Doerig C, Scherf A (2007). A novel protein kinase family in Plasmodium falciparum is differentially transcribed and secreted to various cellular compartments of the host cell. Mol. Microbiol.

[68] Davies H (2020). An exported kinase family mediates species-specific erythrocyte remodelling and virulence in human malaria. Nat. Microbiol..

[69] Maier AG (2007). Skeleton-binding protein 1 functions at the parasitophorous vacuole membrane to traffic PfEMP1 to the Plasmodium falciparum-infected erythrocyte surface. Blood.

[70] Ravetch JV, Kochan J, Perkins M (1985). Isolation of the gene for a glycophorin-binding protein implicated in erythrocyte invasion by a malaria parasite. Science.

[71] Kato K, Sudo A, Kobayashi K, Tohya Y, Akashi H (2008). Characterization of Plasmodium falciparum protein kinase 2. Mol. Biochem. Parasitol..

[72] Wang, W. et al. A Thioredoxin Homologous Protein of Plasmodium falciparum Participates in Erythrocyte Invasion. *Infect Immun* **86**. 10.1128/IAI.00289-18 (2018) 10.1128/IAI.00289-18 PMC6056854 29844242

[73] Kumar K, Singal A, Rizvi MM, Chauhan VS (2008). High mobility group box (HMGB) proteins of Plasmodium falciparum: DNA binding proteins with pro-inflammatory activity. Parasitol. Int.

[74] Spillman, N. J., Dalmia, V. K. & Goldberg, D. E. Exported Epoxide Hydrolases Modulate Erythrocyte Vasoactive Lipids during Plasmodium falciparum Infection. *mBio* **7**. 10.1128/mBio.01538-16 (2016) 10.1128/mBio.01538-16 PMC5082902 27795395

[75] Kanehisa M, Goto S (2000). KEGG: kyoto encyclopedia of genes and genomes. Nucleic Acids Res..

[76] Kanehisa M, Furumichi M, Sato Y, Kawashima M, Ishiguro-Watanabe M (2023). KEGG for taxonomy-based analysis of pathways and genomes. Nucleic Acids Res..

[77] Kanehisa M (2019). Toward understanding the origin and evolution of cellular organisms. Protein Sci..

[78] Luo W, Pant G, Bhavnasi YK, Blanchard SG, Brouwer C (2017). Pathview web: user friendly pathway visualization and data integration. Nucleic Acids Res..

[79] Luo W, Brouwer C (2013). Pathview: an R/Bioconductor package for pathway-based data integration and visualization. Bioinformatics.

[80] Jin Z, Mendu SK, Birnir B (2013). GABA is an effective immunomodulatory molecule. Amino Acids.

[81] Esau C (2001). Deletion of calcineurin and myocyte enhancer factor 2 (MEF2) binding domain of Cabin1 results in enhanced cytokine gene expression in T cells. J. Exp. Med..

[82] Di Giorgio E (2021). A biological circuit involving Mef2c, Mef2d, and Hdac9 controls the immunosuppressive functions of CD4+Foxp3+ T-regulatory cells. Front. Immunol..

[83] Saito S (2021). RASAL3 is a putative RasGAP modulating inflammatory response by neutrophils. Front. Immunol..

[84] Wang C (2002). Modulation of Mac-1 (CD11b/CD18)-mediated adhesion by the leukocyte-specific protein 1 is key to its role in neutrophil polarization and chemotaxis. J. Immunol..

[85] Liu L (2005). LSP1 is an endothelial gatekeeper of leukocyte transendothelial migration. J. Exp. Med..

[86] Hossain M (2015). Endothelial LSP1 modulates extravascular neutrophil chemotaxis by regulating nonhematopoietic vascular PECAM-1 expression. J. Immunol..

[87] Scholzen, A. & Sauerwein, R. W. in *Trends in Parasitology* Vol. 29, 252–262 (Elsevier Ltd, 2013). 10.1016/j.pt.2013.03.002 23562778

[88] Struik SS, Riley EM (2004). Does malaria suffer from lack of memory?. Immunol. Rev..

[89] Achtman, A. H., Bull, P. C., Stephens, R. & Langhorne, J. In *Immunology and Immunopathogenesis of Malaria* (ed Langhorne, J) 71–102 (Springer Berlin Heidelberg, 2005).

[90] Li Y (2019). The regulatory roles of neutrophils in adaptive immunity. Cell Commun. Signal..

[91] Elzey BD (2003). Platelet-mediated modulation of adaptive immunity. A communication link between innate and adaptive immune compartments. Immunity.

[92] Sowa JM, Crist SA, Ratliff TL, Elzey BD (2009). Platelet influence on T- and B-cell responses. Arch. Immunol. Ther. Exp..

[93] Tan S (2022). Platelets enhance CD4+ central memory T cell responses via platelet factor 4-dependent mitochondrial biogenesis and cell proliferation. Platelets.

[94] Vono M (2017). Neutrophils acquire the capacity for antigen presentation to memory CD4(+) T cells in vitro and ex vivo. Blood.

[95] Soon MSF (2020). Transcriptome dynamics of CD4(+) T cells during malaria maps gradual transit from effector to memory. Nat. Immunol..

[96] Kurup SP, Butler NS, Harty JT (2019). T cell-mediated immunity to malaria. Nat. Rev. Immunol..

[97] Zander RA (2017). Th1-like Plasmodium-specific memory CD4(+) T cells support humoral immunity. Cell Rep..

[98] Subramanian A (2005). Gene set enrichment analysis: a knowledge-based approach for interpreting genome-wide expression profiles. Proc. Natl Acad. Sci. USA.

[99] Dustin ML (2007). Cell adhesion molecules and actin cytoskeleton at immune synapses and kinapses. Curr. Opin. Cell Biol..

[100] Kumari S, Curado S, Mayya V, Dustin ML (2014). T cell antigen receptor activation and actin cytoskeleton remodeling. Biochim. Biophys. Acta.

[101] Yuseff MI, Lankar D, Lennon-Dumenil AM (2009). Dynamics of membrane trafficking downstream of B and T cell receptor engagement: impact on immune synapses. Traffic.

[102] Janeway, C., Travers, P., Walport, M. & Shlomchik, M.J. In *Immunobiology: The Immune System in Heath and Disease*. (Garland Science, 2001).

[103] Tran T (2010). Interferonbeta-1b induces the expression of RGS1 a negative regulator of G-protein signaling. Int. J. Cell Biol..

[104] Merlo LMF (2020). Differential roles of IDO1 and IDO2 in T and B cell inflammatory immune responses. Front. Immunol..

[105] Metzemaekers M, Vanheule V, Janssens R, Struyf S, Proost P (2017). Overview of the mechanisms that may contribute to the non-redundant activities of interferon-inducible CXC Chemokine Receptor 3 ligands. Front Immunol..

[106] Gowda DC, Wu X (2018). Parasite recognition and signaling mechanisms in innate immune responses to malaria. Front. Immunol..

[107] Clay GM, Sutterwala FS, Wilson ME (2014). NLR proteins and parasitic disease. Immunol. Res.

[108] He X, Xia L, Tumas KC, Wu J, Su XZ (2020). Type I interferons and malaria: a double-edge sword against a complex parasitic disease. Front. Cell Infect. Microbiol.

[109] Pishesha N, Harmand TJ, Ploegh HL (2022). A guide to antigen processing and presentation. Nat. Rev. Immunol..

[110] Baird JK (2003). Adult Javanese migrants to Indonesian Papua at high risk of severe disease caused by malaria. Epidemiol. Infect..

[111] Baird JK (2003). Onset of clinical immunity to Plasmodium falciparum among Javanese migrants to Indonesian Papua. Ann. Trop. Med Parasitol..

[112] Langhorne J, Ndungu FM, Sponaas AM, Marsh K (2008). Immunity to malaria: more questions than answers. Nat. Immunol..

[113] Rodriguez-Barraquer I (2016). Quantifying heterogeneous malaria exposure and clinical protection in a cohort of Ugandan children. J. Infect. Dis..

[114] Henry NB (2019). Biology of Plasmodium falciparum gametocyte sex ratio and implications in malaria parasite transmission. Malar. J..

[115] Carter LM (2013). Stress and sex in malaria parasites: why does commitment vary?. Evol. Med. Public Health.

[116] Bousema T, Drakeley C (2011). Epidemiology and infectivity of Plasmodium falciparum and Plasmodium vivax gametocytes in relation to malaria control and elimination. Clin. Microbiol. Rev..

[117] Sowunmi A, Balogun ST, Gbotosho GO, Happi CT (2009). Plasmodium falciparum gametocyte sex ratios in symptomatic children treated with antimalarial drugs. Acta Trop..

[118] Sowunmi A, Gbotosho GO, Happi CT, Folarin OA, Balogun ST (2009). Population structure of Plasmodium falciparum gametocyte sex ratios in malarious children in an endemic area. Parasitol. Int..

[119] Mitri C, Thiery I, Bourgouin C, Paul RE (2009). Density-dependent impact of the human malaria parasite Plasmodium falciparum gametocyte sex ratio on mosquito infection rates. Proc. Biol. Sci..

[120] Walldorf JA (2015). School-age children are a reservoir of malaria infection in Malawi. PLoS One.

[121] Coalson JE (2018). Simulation models predict that school-age children are responsible for most human-to-mosquito Plasmodium falciparum transmission in southern Malawi. Malar. J..

[122] White M, Watson J (2018). Age, exposure and immunity. Elife.

[123] Piganis RA (2011). Suppressor of cytokine signaling (SOCS) 1 inhibits type I interferon (IFN) signaling via the interferon alpha receptor (IFNAR1)-associated tyrosine kinase Tyk2. J. Biol. Chem..

[124] Matsumiya T, Stafforini DM (2010). Function and regulation of retinoic acid-inducible gene-I. Crit. Rev. Immunol..

[125] Peng, Y. C. et al. Plasmodium yoelii erythrocyte-binding-like protein modulates host cell membrane structure, immunity, and disease severity. *mBio* **11**. 10.1128/mBio.02995-19 (2020) 10.1128/mBio.02995-19 PMC6946805 31911494

[126] Le Bon A (2001). Type i interferons potently enhance humoral immunity and can promote isotype switching by stimulating dendritic cells in vivo. Immunity.

[127] Ofir-Birin Y (2021). Malaria parasites both repress host CXCL10 and use it as a cue for growth acceleration. Nat. Commun..

[128] Long GH, Chan BH, Allen JE, Read AF, Graham AL (2008). Blockade of TNF receptor 1 reduces disease severity but increases parasite transmission during Plasmodium chabaudi chabaudi infection. Int. J. Parasitol..

[129] Long GH, Chan BH, Allen JE, Read AF, Graham AL (2008). Experimental manipulation of immune-mediated disease and its fitness costs for rodent malaria parasites. BMC Evolut. Biol..

[130] Kim D, Paggi JM, Park C, Bennett C, Salzberg SL (2019). Graph-based genome alignment and genotyping with HISAT2 and HISAT-genotype. Nat. Biotechnol..

[131] Aurrecoechea C (2009). PlasmoDB: a functional genomic database for malaria parasites. Nucleic Acids Res..

[132] Liao Y, Smyth GK, Shi W (2014). featureCounts: an efficient general purpose program for assigning sequence reads to genomic features. Bioinformatics.

[133] Robinson MD, McCarthy DJ, Smyth G (2010). K. edgeR: a Bioconductor package for differential expression analysis of digital gene expression data. Bioinformatics.

[134] Benjamini Y, Hochberg Y (1995). Controlling the false discovery rate: a practical and powerful approach to multiple testing.. J. R. Stat. Soc..

[135] Howick, V. M. et al. The malaria cell atlas: single parasite transcriptomes across the complete Plasmodium life cycle. *Science* **365**. 10.1126/science.aaw2619 (2019) 10.1126/science.aaw2619 PMC7056351 31439762

[136] Van der Auwera, G. A. & O’Connor, B. D. *Genomics in the Cloud: Using Docker, GATK, and WDL in Terra* 1st edn, (O’Reilly Media, 2020).

[137] Lee, S. & Bahlo, M. moimix: an R package for assessing clonality in high-througput sequencing data. 10.5281/zenodo.58257 (2016).

[138] Tebben, K. Gene expression analyses reveal differences in children’s response to malaria according to their age. 10.5281/zenodo.10631324 (2023). 10.1038/s41467-024-46416-3 PMC10918175 38448421

