# Supplementary material for: Gene expression analyses reveal differences in children’s response to malaria according to their age
Source: Nat Commun. 2024 Mar 6;15:2021. doi: 10.1038/s41467-024-46416-3 (PMC10918175; doi:10.1038/s41467-024-46416-3)
Supplement: Supplementary file 1 — Supplementary Information [file 41467_2024_46416_MOESM1_ESM.pdf]

Supplementary Figures and Legends:

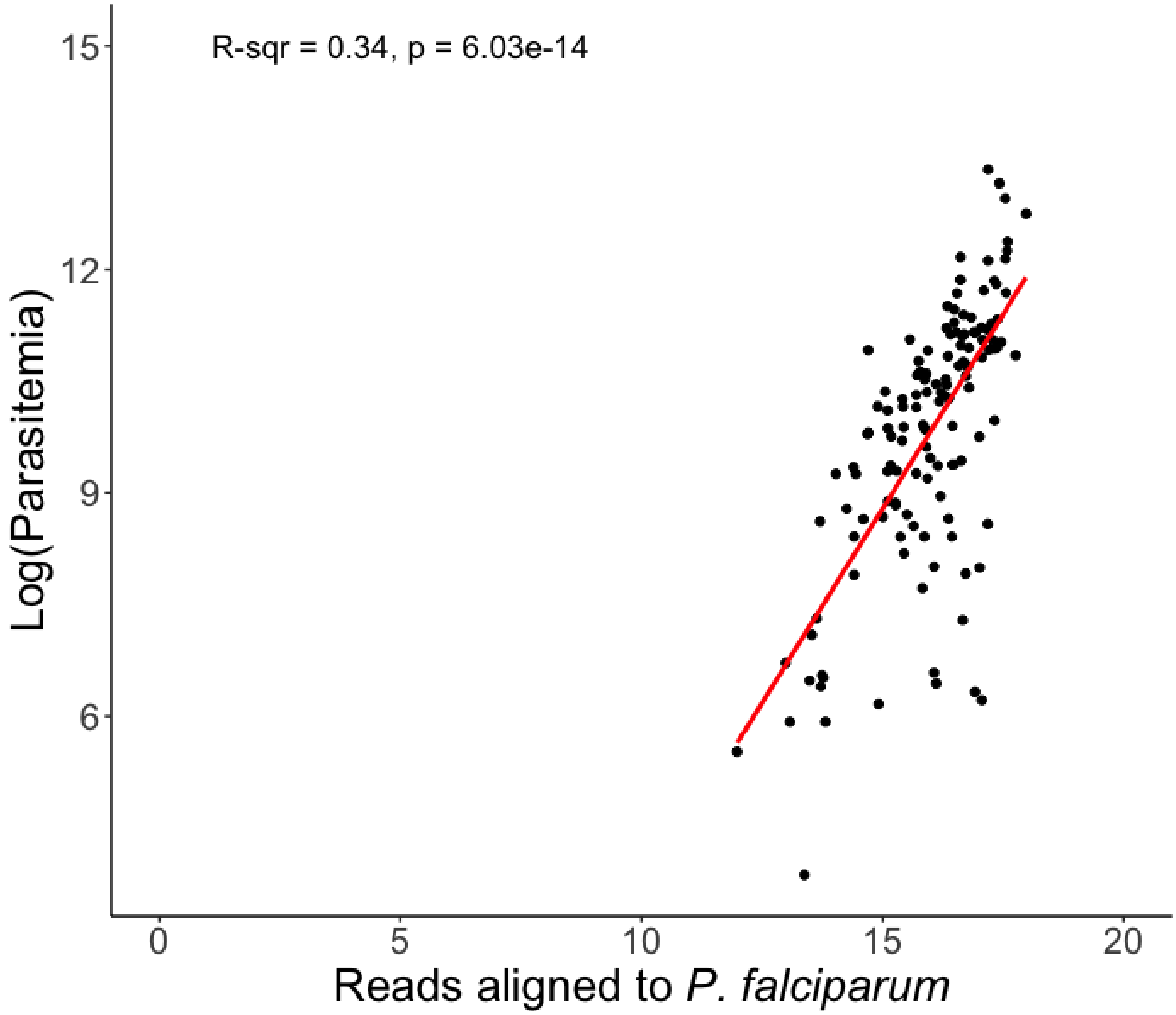

Supplemental Figure 1: Parasitemia and mapped reads are highly correlated.

Association of the the log of *P. falciparum* parasitemia measured by microscopy (y) vs. log of number of reads mapping to *P. falciparum* (x) measured by linear regression ($p = 6.03 \times 10^{-14}$). ($n = 136$ individuals)

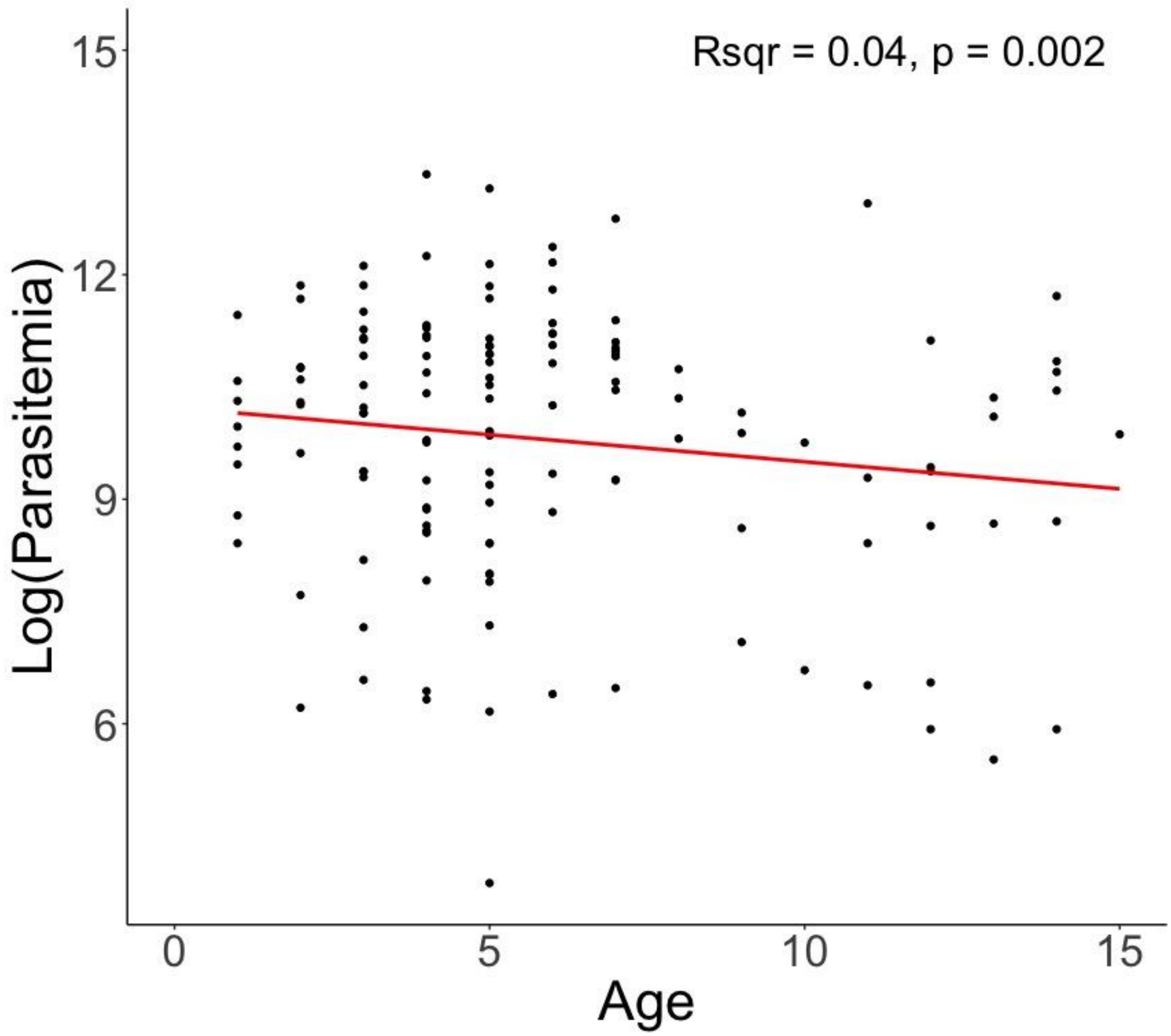

Supplemental Figure 2: Age and parasitemia are weakly correlated, with older children having lower parasitemia. Association of age in years (y) with the log of the *P. falciparum* parasitemia measured by microscopy measured by linear regression (p = 0.002). (n = 136 individuals)

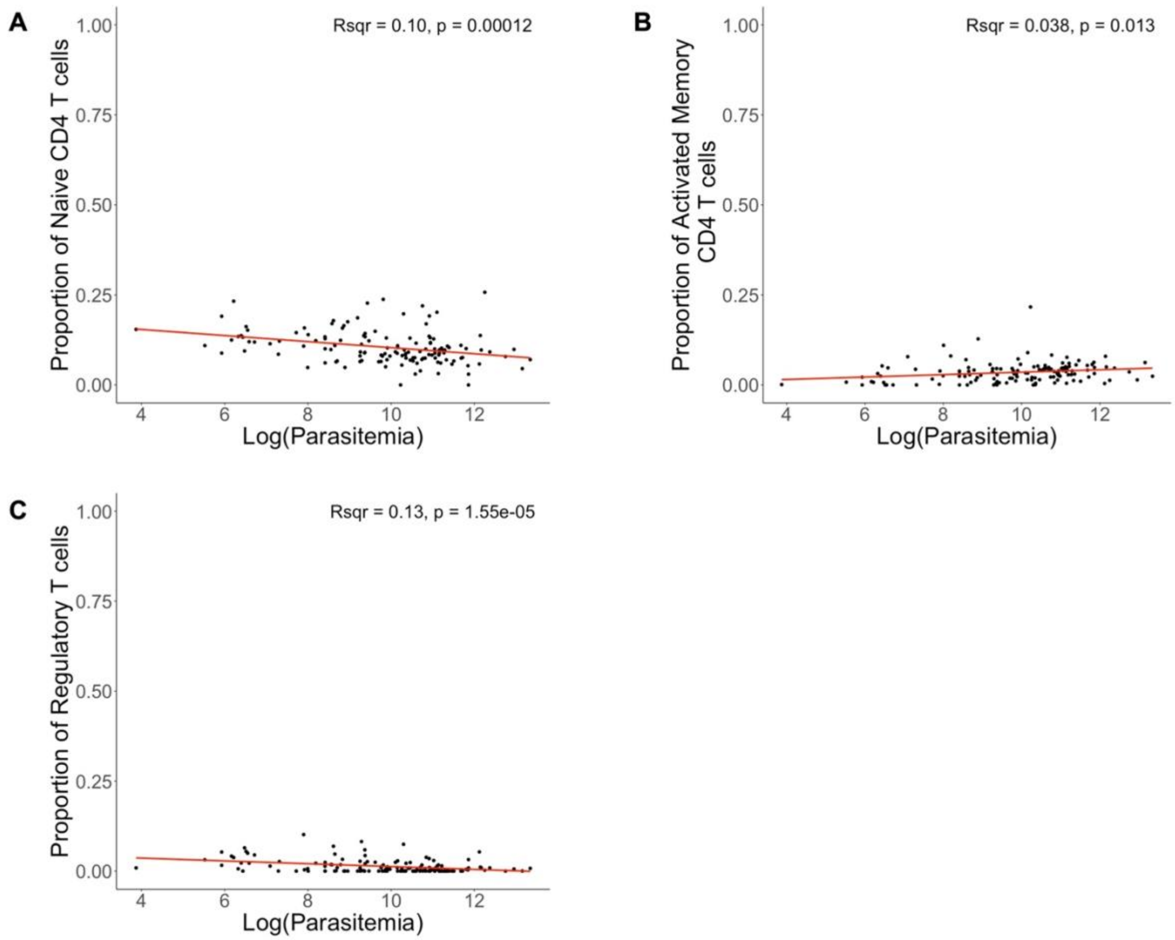

Supplemental Figure 3: The proportion of specific T cell subsets (y), estimated by gene expression deconvolution that are correlated with the log of the *P. falciparum* parasitemia measured by microscopy (x). Proportion of naïve CD4+ T cells (p = 0.00012) **(A)** activated CD4+ memory T cells (p = 0.013) **(B)** and regulatory T cells (p = 1.55 x 10^{-5}) **(C)** correlated with log(parasitemia) measured by linear regression. (n = 136 individuals)

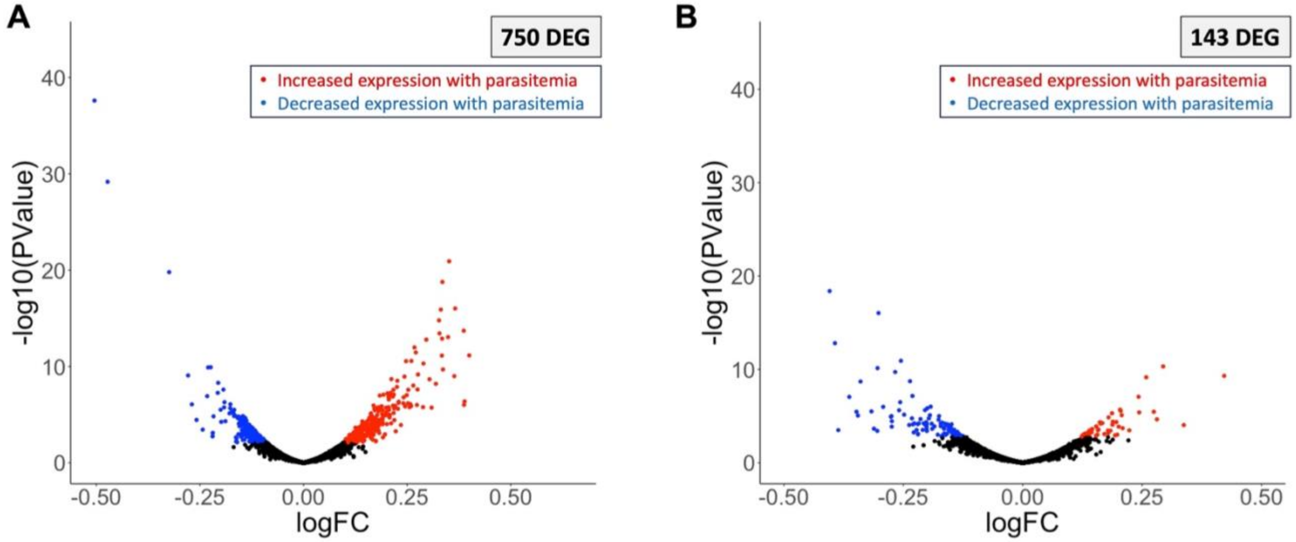

Supplemental Figure 4: Some human genes are correlated with parasitemia, separate from age. Volcano plot of human genes associated with log(parasitemia) in four- to five-year-old children using a quasi-likelihood negative binomial model and correcting for multiple testing using FDR (FDR = 0.1). Each point represents one gene, plotted by its P-value (y) and log(fold-change) value (x). Blue points are genes that are significantly negatively associated with log(parasitemia). Red points are genes that are significantly positively associated with log(parasitemia). **A)** Model unadjusted for immune cell composition; 750 significant genes. **B)** Model adjusted for immune cell composition; 143 significant genes*DEG = differentially expressed gene.

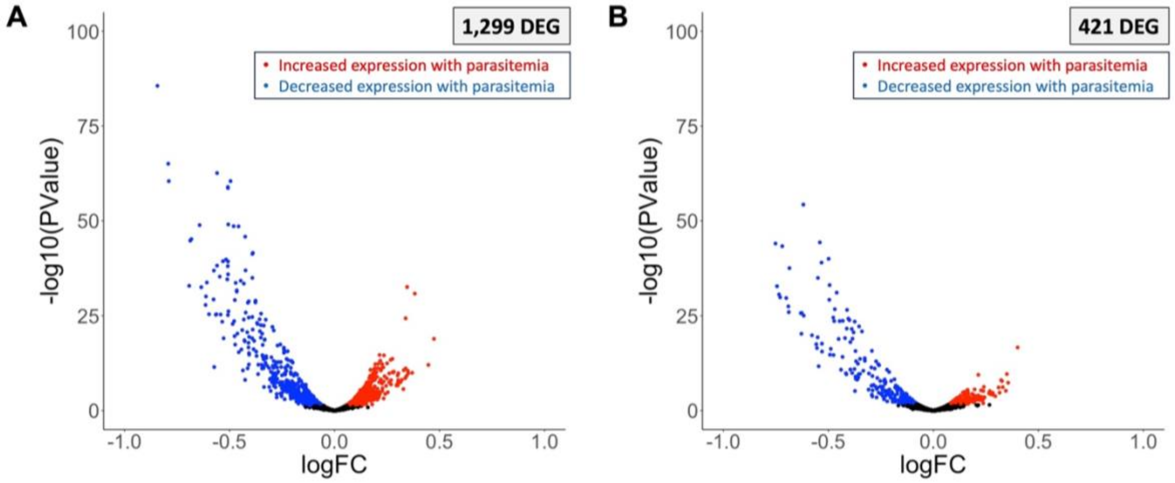

Supplemental Figure 5: Some parasite genes are correlated with parasitemia, separate from age. Volcano plot of *P. falciparum* genes associated with log(parasitemia) in four- to five-year-old children using a quasi-likelihood negative binomial model, correcting for multiple testing using FDR (FDR = 0.1). Each point represents one gene, plotted by its P-value (y) and log(fold-change) value (x). Blue points are genes that are significantly negatively associated with log(parasitemia). Red points are genes that are significantly positively associated with log(parasitemia). **A)** Model unadjusted for developmental stage composition; 1,299 significant genes. **B)** Model adjusted for developmental stage composition; 421 significant genes. *DEG = differentially expressed gene.

A

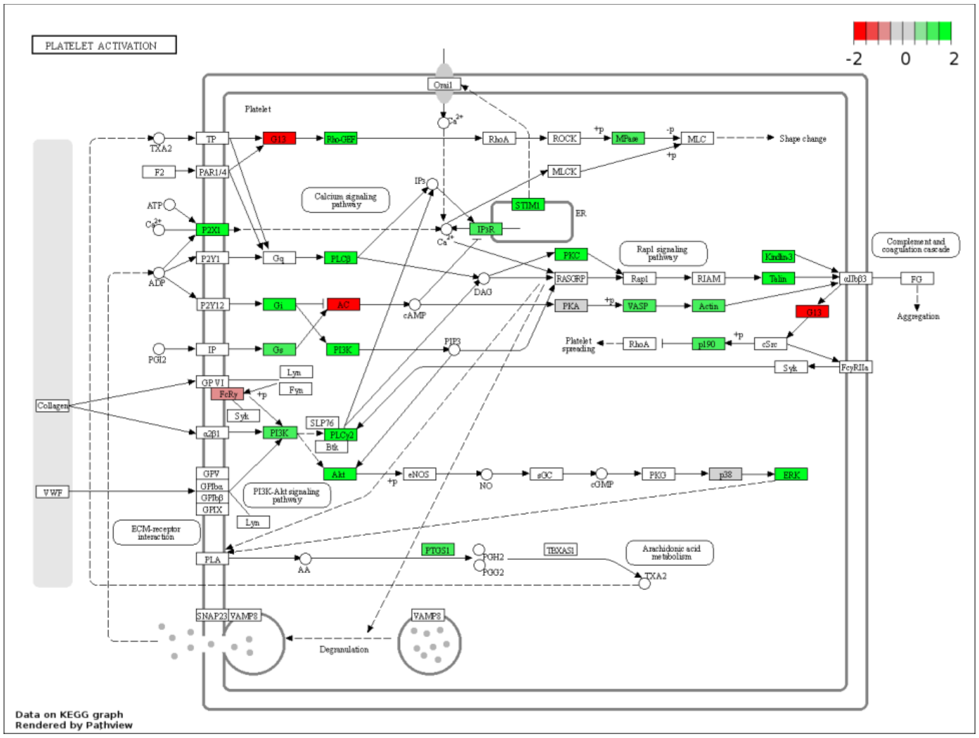
PLATELET ACTIVATION
-2
0
2
Orai1
Platelet
TXA2
TP
G13
Rho-GEF
RhoA
ROCK
MPase
MLC
Shape change
F2
PAR1/4
MLCK
IP3
Calcium signaling pathway
STIM1
ER
ATP
P2X1
IP3R
P2Y1
Gq
PLCβ
PKC
Rap1 signaling pathway
Kindlin-3
Complement and coagulation cascade
ADP
DAG
RASGRP
Rap1
RIAM
Talin
αIIbβ3
FG
Aggregation
P2Y12
Gi
AC
cAMP
PKA
VASP
Actin
G13
PGI2
IP
Gs
PI3K
PIP3
Platelet spreading
RhoA
p190
cSrc
GP VI
Lyn
Syk
FcγRIIa
Collagen
FcRγ
Fyn
Syk
SLP76
α2β1
PI3K
PLCγ2
Btk
Akt
eNOS
NO
sGC
cGMP
PKG
p38
ERK
GPV
GPIbα
GPIbβ
GPIX
PI3K-Akt signaling pathway
VWF
Lyn
ECM-receptor interaction
PTGS1
TBXAS1
Arachidonic acid metabolism
PLA
AA
PGH2
PGG2
TXA2
SNAP23
VAMP8
VAMP8
Degranulation
Data on KEGG graph
Rendered by Pathview

B

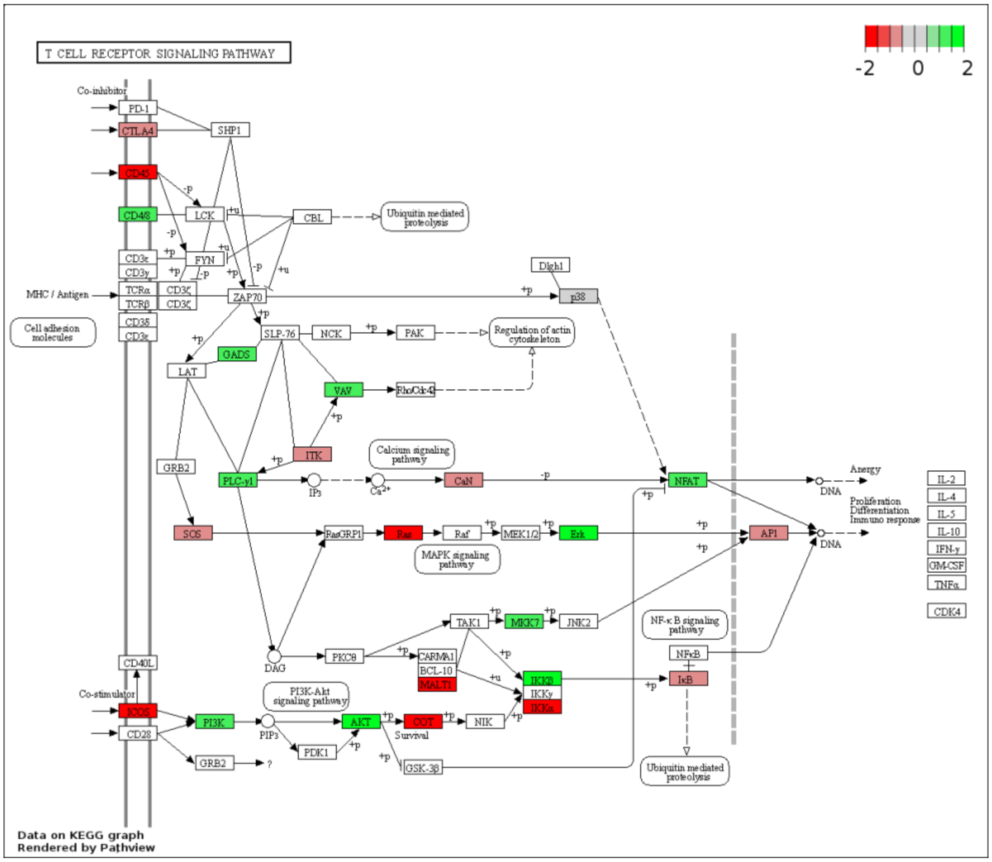
T CELL RECEPTOR SIGNALING PATHWAY
-2
0
2
Co-inhibitor
PD-1
CTLA4
SHP1
CD45
LCK
CBL
Ubiquitin mediated proteolysis
CD4/8
CD3ε
CD3γ
FYN
Dlgh1
MHC / Antigen
TCRα
TCRβ
CD3ζ
CD3ζ
ZAP70
p38
CD3δ
CD3ε
Cell adhesion molecules
SLP-76
NCK
PAK
Regulation of actin cytoskeleton
GADS
LAT
VAV
RhoCdc42
ITK
Calcium signaling pathway
GRB2
PLC-γ1
IP3
Ca2+
CaN
NFAT
Anergy
DNA
Proliferation
Differentiation
Immuno response
SOS
RasGRP1
Ras
Raf
MEK1/2
Erk
AP1
DNA
MAPK signaling pathway
IL-2
IL-4
IL-5
IL-10
IFN-γ
GM-CSF
TNFα
CDK4
TAK1
MKK7
JNK2
NF-κB signaling pathway
DAG
PKCθ
CARMA1
BCL-10
MALT1
NFκB
IKKβ
IKKγ
IKKα
IκB
CD40L
Co-stimulator
ICOS
PI3K
PI3K-Akt signaling pathway
AKT
COT
NIK
CD28
PIP3
PDK1
Survival
GRB2
GSK-3β
Ubiquitin mediated proteolysis
Data on KEGG graph
Rendered by Pathview

C

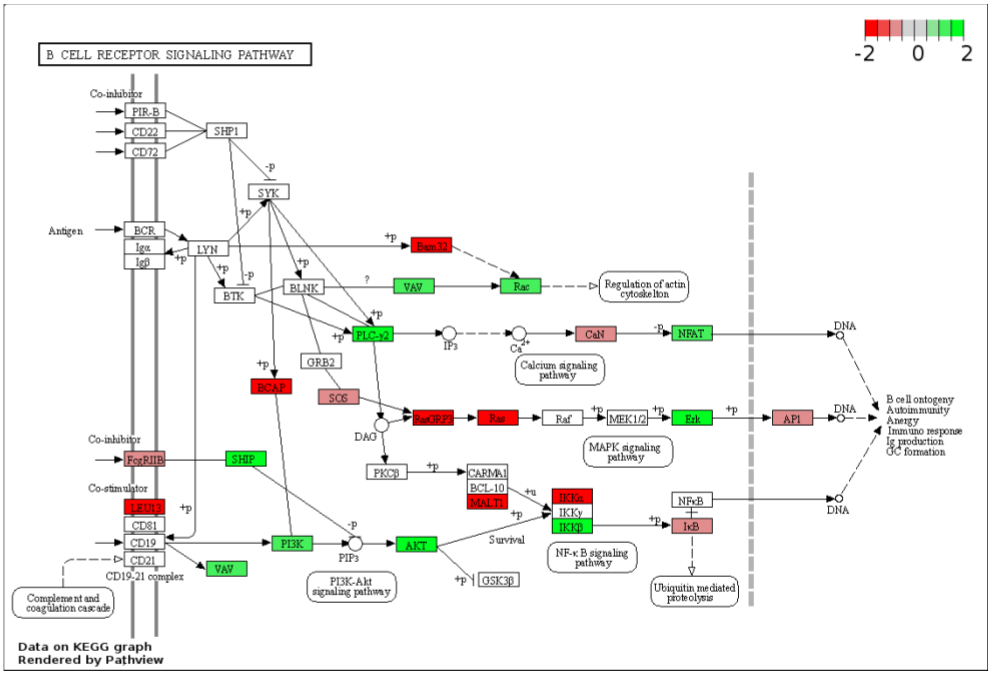
B CELL RECEPTOR SIGNALING PATHWAY
-2 0 2
Co-inhibitor
PIR-B
CD22
CD72
SHP1
SYK
Antigen
BCR
Igα
Igβ
LYN
BTK
BLNK
Bam32
VAV
Rac
Regulation of actin cytoskelton
PLC-γ2
IP3
Ca2+
CaN
NFAT
DNA
Calcium signaling pathway
GRB2
BCAP
SOS
DAG
RasGRP3
Ras
Raf
MEK1/2
Erk
AP1
MAPK signaling pathway
B cell ontogeny
Autoimmunity
Anergy
Immuno response
Ig production
GC formation
FcγRIIB
SHIP
PKCβ
CARMA1
BCL-10
MALT1
IKKα
IKKγ
IKKβ
NFκB
IκB
Co-stimulator
LEU13
CD81
CD19
CD21
CD19-21 complex
PI3K
PIP3
AKT
Survival
GSK3β
NF-κB signaling pathway
Ubiquitin mediated proteolysis
PI3K-Akt signaling pathway
Complement and coagulation cascade
Data on KEGG graph
Rendered by Pathview

D

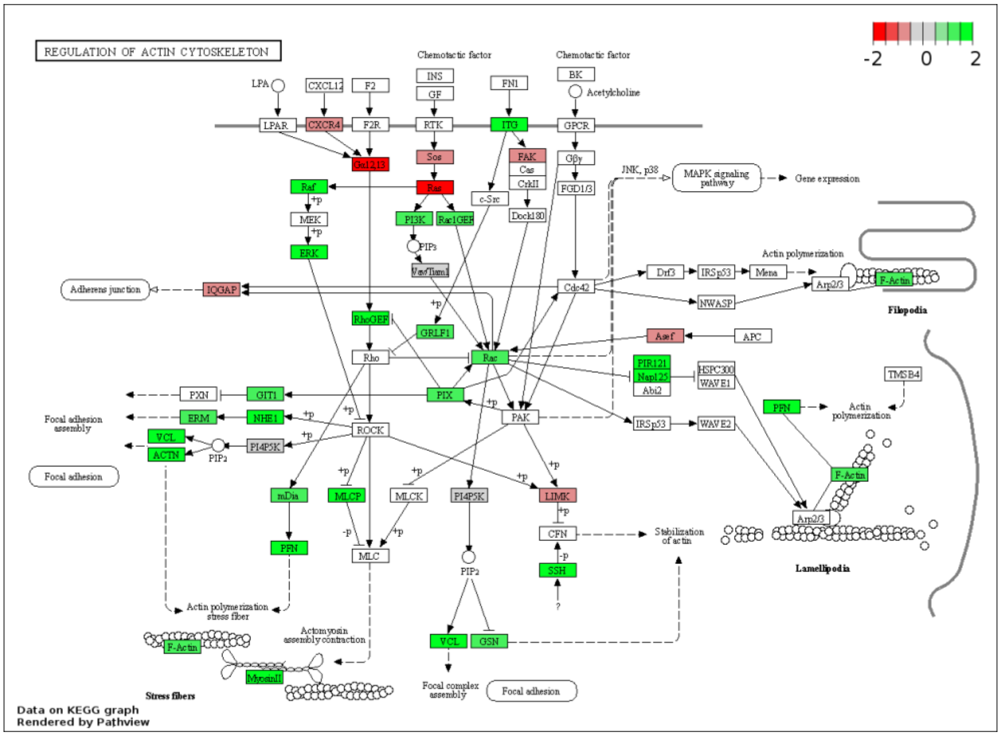
REGULATION OF ACTIN CYTOSKELETON
-2 0 2
Chemotactic factor
Chemotactic factor
LPA
CXCL12
F2
INS
GF
FN1
BK
Acetylcholine
LPAR
CXCR4
F2R
RTK
ITG
GPCR
Gα12/13
Sos
FAK
Cas
CrkII
Gβγ
FGD1/3
Raf
Ras
c-Src
MEK
PI3K
Rac1GEF
Dock180
ERK
PIP3
Vav/Tiam1
JNK, p38
MAPK signaling pathway
Gene expression
Actin polymerization
Adherens junction
IQGAP
Cdc42
Drf3
IRSp53
Mena
Arp2/3
F-Actin
NWASP
Filopodia
RhoGEF
GRLF1
Asef
APC
Rho
Rac
PIR121
Nap125
Abi2
HSPC300
WAVE1
TMSB4
PXN
GIT1
PIX
PAK
PFN
Actin polymerization
Focal adhesion assembly
ERM
NHE1
ROCK
IRSp53
WAVE2
VCL
ACTN
PIP2
PI4P5K
Focal adhesion
mDia
MLCP
MLCK
PI4P5K
LIMK
F-Actin
Arp2/3
CFN
Stabilization of actin
Lamellipodia
PFN
MLC
SSH
PIP2
Actin polymerization stress fiber
Actomyosin assembly contraction
F-Actin
MyosinII
Stress fibers
VCL
GSN
Focal complex assembly
Focal adhesion
Data on KEGG graph
Rendered by Pathview

E

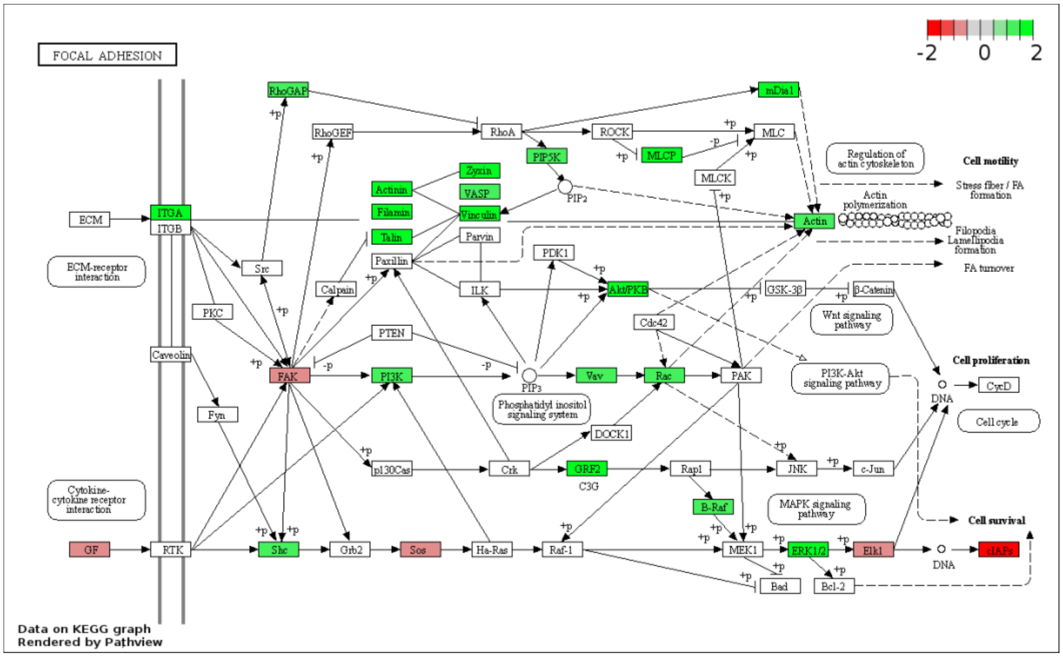

Supplemental Figure 6: Human genes that were significantly positively associated with host age at the sequenced infection, mapped onto KEGG Pathways. Green boxes represent genes that are significantly positively and red boxes represent genes that are significantly negatively associated with age at FDR = 0.1. To visualize genes that did change expression in our analysis, but did not meet our significance threshold (FDR = 0.1), light green boxes represent genes that increased in expression with age and light red boxes represent genes that decreased in expression with age at FDR = 0.25. A) Platelet activation pathway **B)** T cell receptor signaling pathway **C)** B cell receptor signaling pathway **D)** Regulation of the actin cytoskeleton pathway **E)** Focal adhesion pathway.

A

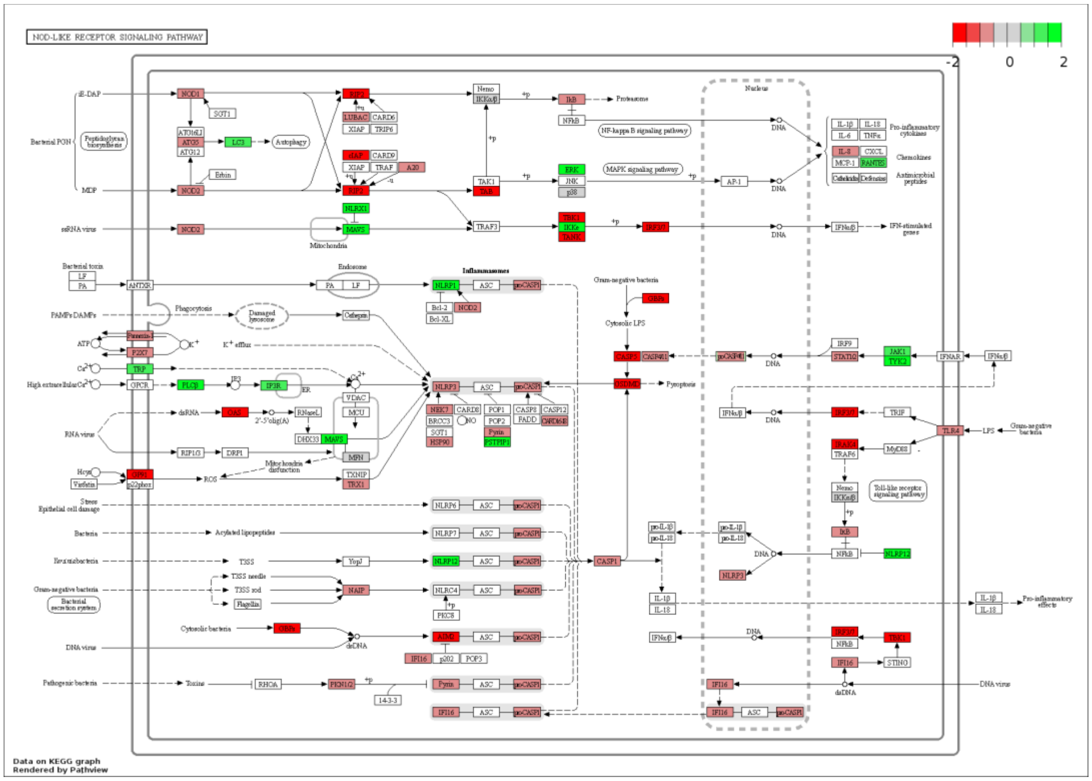
NOD-LIKE RECEPTOR SIGNALING PATHWAY
-2
0
2
Data on KEGG graph
Rendered by Pathview

B

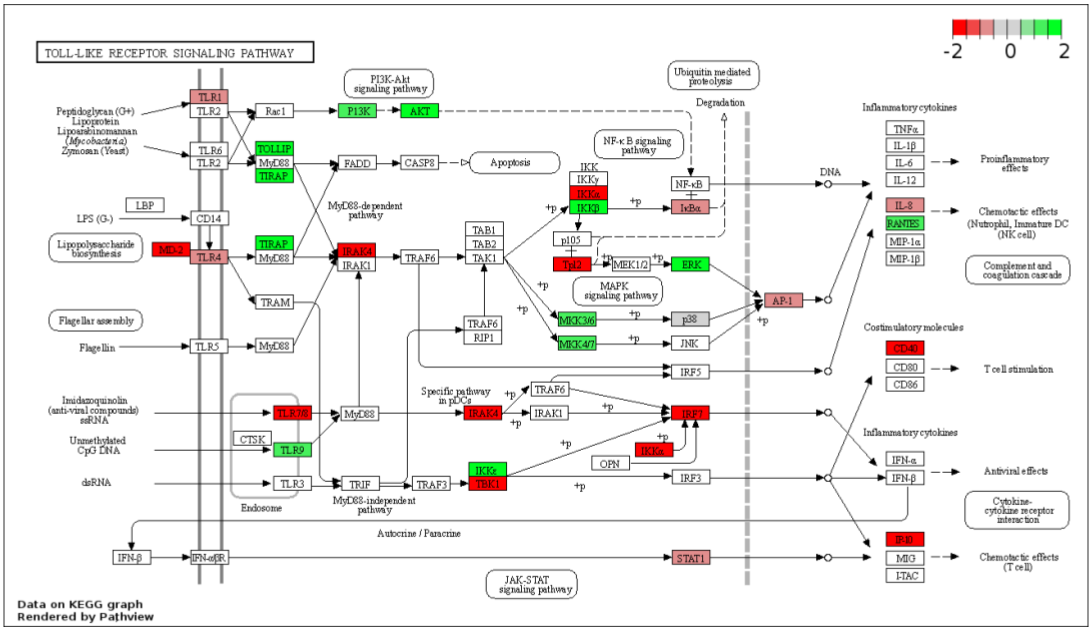
TOLL-LIKE RECEPTOR SIGNALING PATHWAY
-2
0
2
Peptidoglycan (G+)
Lipoprotein
Lipoarabinomannan
(Mycobacteria)
Zymosan (Yeast)
LPS (G-)
LBP
CD14
Lipopolysaccharide biosynthesis
Flagellar assembly
Flagellin
Imidazoquinolin
(anti-viral compounds)
ssRNA
Unmethylated CpG DNA
dsRNA
TLR1
TLR2
TLR6
TLR4
MD-2
TLR5
TLR7/8
TLR9
TLR3
CTSK
Endosome
Rac1
PI3K
AKT
PI3K-Akt signaling pathway
TOLLIP
MyD88
TIRAP
TRAM
FADD
CASP8
Apoptosis
MyD88-dependent pathway
IRAK4
IRAK1
TRAF6
TAB1
TAB2
TAK1
TRAF6
RIP1
TRIF
TRAF3
MyD88-independent pathway
Specific pathway in pDCs
IKK
IKKγ
IKKα
IKKβ
IKKε
TBK1
OPN
p105
Tpl2
MEK1/2
ERK
MKK3/6
MKK4/7
p38
JNK
MAPK signaling pathway
NF-κB signaling pathway
NF-κB
IκBα
Ubiquitin mediated proteolysis
Degradation
AP-1
IRF5
IRF7
IRF3
DNA
+p
Autocrine / Paracrine
IFN-β
IFN-α/βR
STAT1
JAK-STAT signaling pathway
Inflammatory cytokines
TNFα
IL-1β
IL-6
IL-12
Proinflammatory effects
IL-8
RANTES
MIP-1α
MIP-1β
Chemotactic effects
(Nutrophil, Immature DC
(NK cell)
Complement and coagulation cascade
Costimulatory molecules
CD40
CD80
CD86
T cell stimulation
Inflammatory cytokines
IFN-α
IFN-β
Antiviral effects
Cytokine-cytokine receptor interaction
IP-10
MIG
I-TAC
Chemotactic effects
(T cell)
Data on KEGG graph
Rendered by Pathview

C

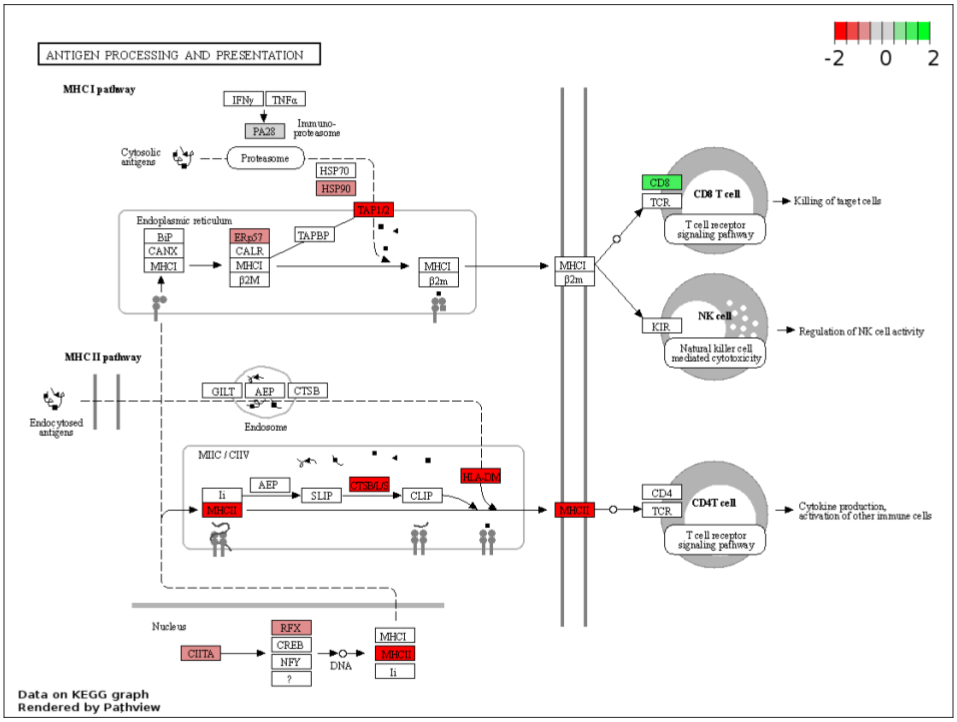
ANTIGEN PROCESSING AND PRESENTATION
-2 0 2
MHC I pathway
MHC II pathway
Endoplasmic reticulum
Endosome
MIIC / CIIV
Nucleus
CD8 T cell
NK cell
CD4T cell
Killing of target cells
Regulation of NK cell activity
Cytokine production, activation of other immune cells
Data on KEGG graph
Rendered by Pathview

D

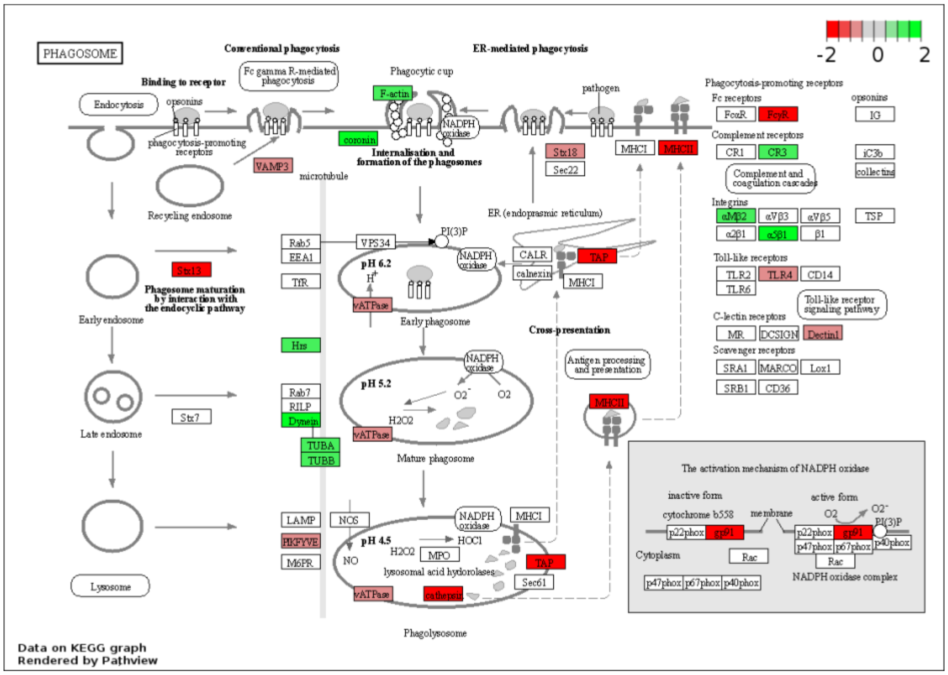
PHAGOSOME
-2 0 2
Conventional phagocytosis
ER-mediated phagocytosis
Phagocytosis-promoting receptors
Early phagosome
Mature phagosome
Phagolysosome
The activation mechanism of NADPH oxidase
Data on KEGG graph
Rendered by Pathview

Supplemental Figure 7: Human genes that were negatively significantly associated with age at the sequenced infection, mapped onto KEGG Pathways. Green boxes represent genes that are significantly positively- and red boxes represent genes that are significantly negatively associated with age at FDR = 0.1. To visualize genes that did change expression in our analysis, but did not meet our significance threshold (FDR = 0.1), light green boxes represent genes that increased in expression with age and light red boxes represent genes that decreased in expression with age at FDR = 0.25. A) NOD-like receptor signaling pathway **B)** Toll-like receptor signaling pathway **C)** Antigen processing and presentation pathway **D)** Phagocytosis pathway.

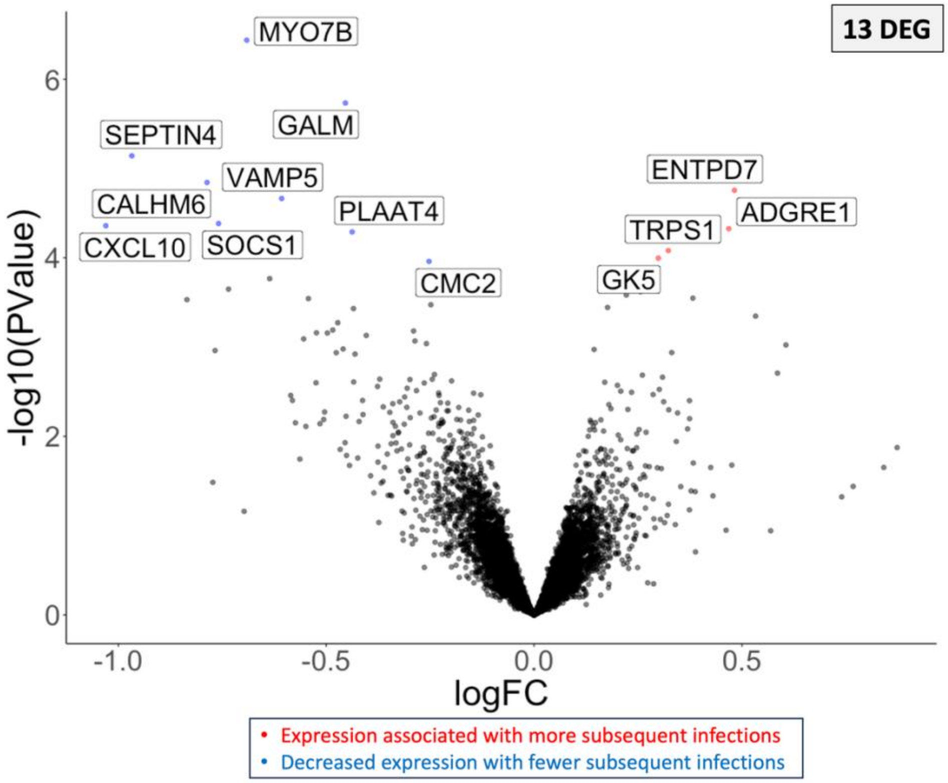

Supplemental Figure 8: Few human genes are associated with the number of subsequent symptomatic infections in the study period. Volcano plot of human genes associated with the number of subsequent symptomatic infections in the study period, weighted for the risk of infection during a given time period, using a quasi-likelihood negative binomial model, correcting for multiple testing using FDR (FDR = 0.1). Each point represents one gene, plotted by its P-value (y) and log(fold-change) value (x). Blue points are genes that are more highly expressed in children who experienced fewer infections. Red points are genes that are more highly expressed in children who experienced more infections. *DEG = differentially expressed gene.

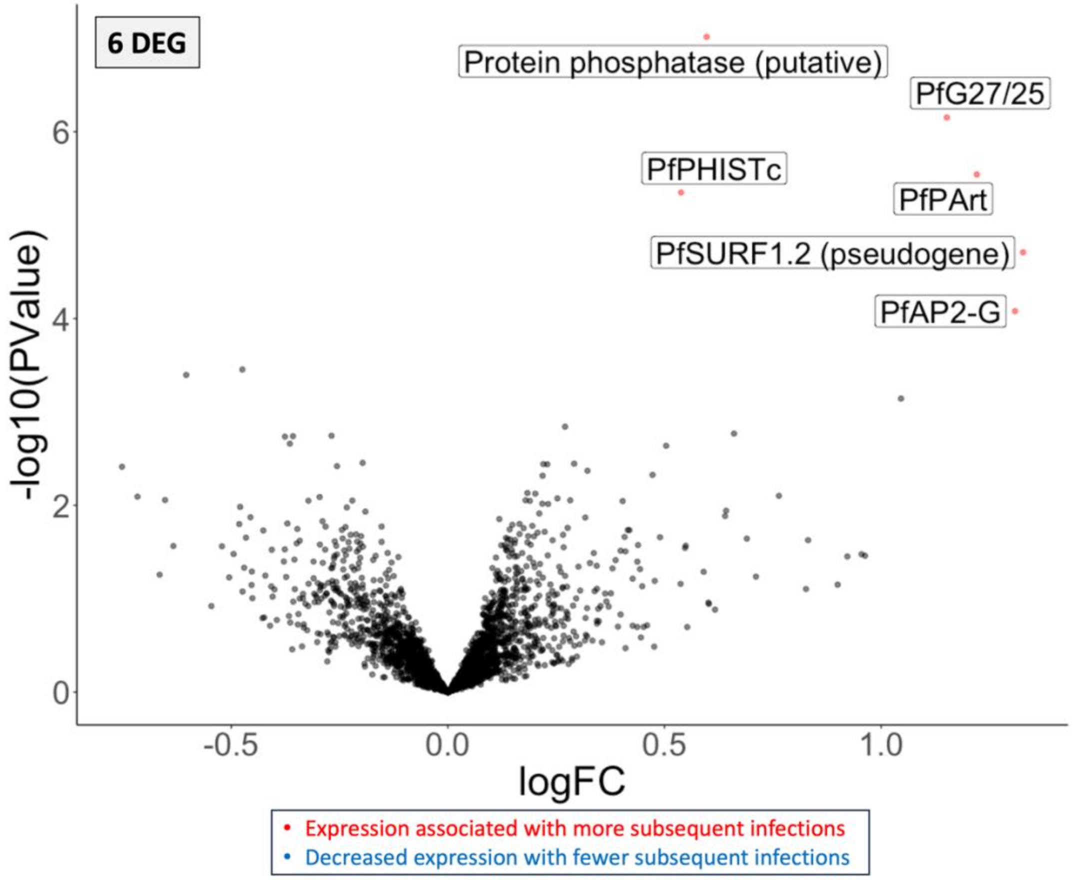

Supplemental Figure 9: Few parasite genes are associated with the number of subsequent symptomatic infections in the study period. Volcano plot of parasite genes associated with the number of subsequent symptomatic infections in the study period, weighted for the risk of infection during a given time period, using a quasi-likelihood negative binomial model, correcting for multiple testing using FDR (FDR = 0.1). Each point represents one gene, plotted by its P-value (y) and log(fold-change) value (x). Blue points are genes that are more highly expressed in parasites infection children who experienced fewer infections. Red points are genes that are more highly expressed in parasites infection children who experienced more infections. Significantly

differentially expressed genes have an (FDR = 0.1). *DEG = differentially expressed gene.

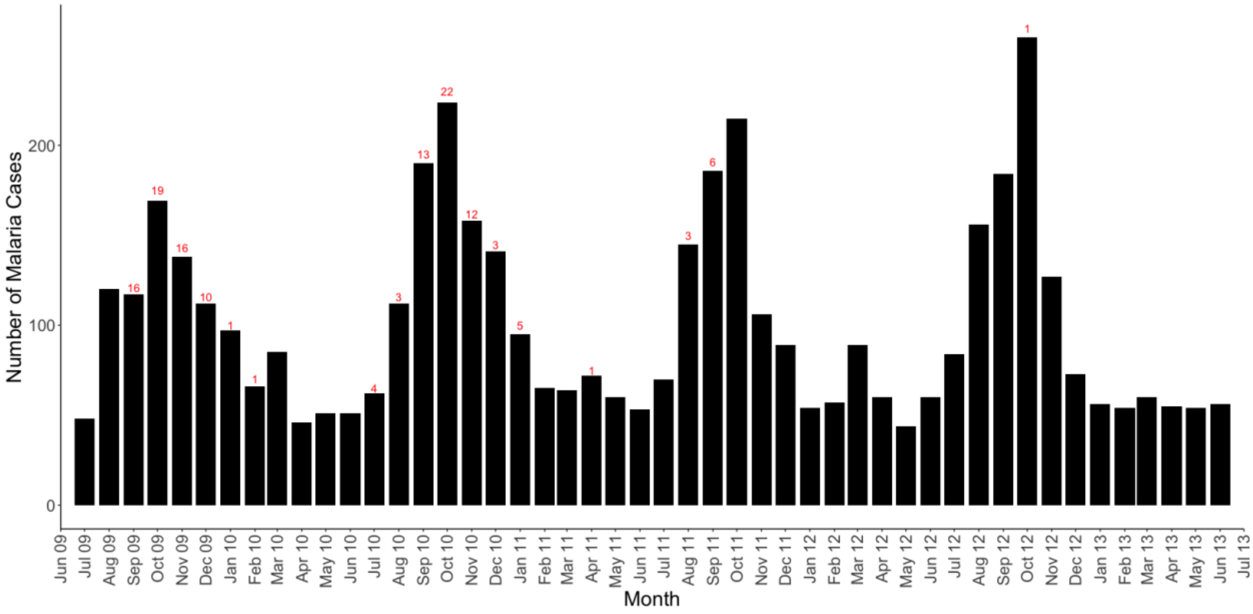

Supplemental Figure 10: Number of samples selected from each month and number of malaria cases each month over the course of a four-year incidence study in Bandiagara, Mali. Each red number depicts the number of infections included in our present study that occurred in that month.
