# Supplementary material for: Gene expression analyses reveal differences in children’s response to malaria according to their age
Source: Nat Commun. 2024 Mar 6;15:2021. doi: 10.1038/s41467-024-46416-3 (PMC10918175; doi:10.1038/s41467-024-46416-3)
Supplement: Supplementary file 3 — Description of Additional Supplementary Files [file 41467_2024_46416_MOESM3_ESM.pdf]

Description of Additional Supplementary Files

File Name: Supplementary Data 1
Description: Sequencing statistics and clinical variables for each individual. Number of reads mapped to each *Plasmodium* species for each individual.

File Name: Supplementary Data 2
Description: Proportion of the variance in expression of each human and *Plasmodium* gene explained by each of the variables in Table 2.

File Name: Supplementary Data 3
Description: Human gene expression correlated with each variable presented in Table 2, both unadjusted and adjusted for cell composition using a quasilikelihood negative binomial generalized model, correcting for multiple testing using false discovery rate (FDR = 0.1).

File Name: Supplementary Data 4
Description: Proportion of human immune cells and *P. falciparum* developmental stages in each individual estimated by CIBERSORTx.

File Name: Supplementary Data 5
Description: Human and *P. falciparum* gene expression correlated with parasitemia at a subset of children aged four to five years old, adjusted for cell composition using a quasi-likelihood negative binomial generalized model, correcting for multiple testing using false discovery rate (FDR = 0.1).

File Name: Supplementary Data 6
Description: *P. falciparum* gene expression correlated with each variable presented in Table 2, both unadjusted and adjusted for cell composition using a quasilikelihood negative binomial generalized model, correcting for multiple testing using false discovery rate (FDR = 0.1).
