# Supplementary material for: Gene expression analyses reveal differences in children’s response to malaria according to their age
Source: Nat Commun. 2024 Mar 6;15:2021. doi: 10.1038/s41467-024-46416-3 (PMC10918175; doi:10.1038/s41467-024-46416-3)
Supplement: Supplementary file 10 — Reporting Summary [file 41467_2024_46416_MOESM10_ESM.pdf]

Corresponding author(s): Kieran Tebben
Last updated by author(s): Nov 6, 2023

Reporting Summary

Nature Portfolio wishes to improve the reproducibility of the work that we publish. This form provides structure for consistency and transparency in reporting. For further information on Nature Portfolio policies, see our Editorial Policies and the Editorial Policy Checklist.

Statistics

For all statistical analyses, confirm that the following items are present in the figure legend, table legend, main text, or Methods section.

n/a	Confirmed	
☐	☒	The exact sample size (n) for each experimental group/condition, given as a discrete number and unit of measurement
☐	☒	A statement on whether measurements were taken from distinct samples or whether the same sample was measured repeatedly
☐	☒	The statistical test(s) used AND whether they are one- or two-sided<br>*Only common tests should be described solely by name; describe more complex techniques in the Methods section.*
☐	☒	A description of all covariates tested
☐	☒	A description of any assumptions or corrections, such as tests of normality and adjustment for multiple comparisons
☐	☒	A full description of the statistical parameters including central tendency (e.g. means) or other basic estimates (e.g. regression coefficient) AND variation (e.g. standard deviation) or associated estimates of uncertainty (e.g. confidence intervals)
☐	☒	For null hypothesis testing, the test statistic (e.g. F, t, r) with confidence intervals, effect sizes, degrees of freedom and P value noted<br>*Give P values as exact values whenever suitable.*
☒	☐	For Bayesian analysis, information on the choice of priors and Markov chain Monte Carlo settings
☐	☒	For hierarchical and complex designs, identification of the appropriate level for tests and full reporting of outcomes
☒	☐	Estimates of effect sizes (e.g. Cohen's d, Pearson's r), indicating how they were calculated

Our web collection on statistics for biologists contains articles on many of the points above.

Software and code

Policy information about availability of computer code

Data collection	No software was used for data collection in this work.
Data analysis	Processing of RNAseq data:<br>Reads were mapped for human and P. falciparum using hisat2 (version 2.1.0).<br>PCR duplicate reads were removed using custom scripts available at https://github.com/tebbenk/symptomatic_malaria (rmdup_general.pl)<br>BAM files were subset to extract only human and only P. falciparum reads using custom scripts available at https://github.com/tebbenk/symptomatic_malaria (subset_bam_human.pl and subset_bam_plasmodiumreads.pl).<br>Count tables were generated from the BAM files using featureCounts (version 1.6.4).<br><br>Calculating complexity of infection:<br>Pre-processing of P. falciparum BAM files for GATK genotyping pipeline from RNA-seq reads was done with custom scripts available at https://github.com/tebbenk/symptomatic_malaria (addRG_splitntrim_symptomatic.pl).<br>Genotype VCF files were generated for all samples using GATK4 HaplotypeCaller.<br>Genotype VCF files were merged using GATK4 GenomicsDBImport and GenotypeGVCFs.<br>VCF files were filtered to include only positions with a mazimum of 2 alleles and no more than 20% missing information using GATK4 VariantFiltration and vcftools (version 0.1.15).<br>Multigene families were removed from the VCF file using custom scripts available at https://github.com/tebbenk/symptomatic_malaria (subset_vcf.pl).<br>Fws was estimated from the final filtered, subset VCF file using moimix (version 0.0.2.9001).

Statistical analyses:
Summary statistics presented in Table 1 were conducted in base R (version 4.0.3).
Differential expression analyses presented in Table 2, Figure 2A/B, Figure 3A/B, Figure 4A/B, Figure 5A/B, Supplemental Figure 4, Supplemental Figure 5, and Supplemental Figure 8 were conducted using Bioconductor EdgeR (version 3.32.1).
Linear regression analyses presented in Figure 2C/D, Figure 3 C/D, Figure 4C/D/E/F, Figure 5C, Supplemental Figure 1, Supplemental Figure 2, and Supplemental Figure 3 were conducted in base R (version 4.0.3).
Analyses of variance in gene expression presented in Figure 1 were conducted with VariancePartition (version 1.20.0).
Gene expression deconvolution from human and plasmodium data was conducted using cibersortx (https://cibersortx.stanford.edu/).
All data visualization was plotted using ggplot2 (version 3.3.6).

For manuscripts utilizing custom algorithms or software that are central to the research but not yet described in published literature, software must be made available to editors and reviewers. We strongly encourage code deposition in a community repository (e.g. GitHub). See the Nature Portfolio guidelines for submitting code & software for further information.

Data

Policy information about availability of data

All manuscripts must include a data availability statement. This statement should provide the following information, where applicable:

- Accession codes, unique identifiers, or web links for publicly available datasets
- A description of any restrictions on data availability
- For clinical datasets or third party data, please ensure that the statement adheres to our policy

All sequence data generated in this study are deposited in the Sequence Read Archive under the BioProject PRJNA962942. Custom scripts are available at https://github.com/tebbenk/symptomatic_malaria. Supplementary Data is available upon publication and at https://github.com/tebbenk/symptomatic_malaria.

Research involving human participants, their data, or biological material

Policy information about studies with human participants or human data. See also policy information about sex, gender (identity/presentation), and sexual orientation and race, ethnicity and racism.

Reporting on sex and gender	Self-reported (by parent) biological sex of each individual child was considered as a covariate in our statistical models for differential expression because of previous reports of sex-based differences in the immune response to malaria. Enrollment in the cohort did not occur on the basis of sex. By chance, we obtained data from 72 male and 64 female children. In our study, we did not find a substantial number of differentially expressed genes between male and female children and biological sex was a very weak contributor to the overall variance in gene expression in our cohort. Consent was collected at the time of enrollment in the cohort to use biological information, such as sex.
Reporting on race, ethnicity, or other socially relevant groupings	Participant ethnicity was collected at enrollment in the cohort because of reported differences in malaria risk between ethnic groups in Mali, with the Fulani ethnic group having far lower risk of malaria than other ethnic groups. Because the majority of the participants in our cohort were of the Dogon ethnic group, with very small sample sizes from other ethnic groups, participant ethnicity was not included as part of our analyses.
Population characteristics	Our cohort includes children from ages 1 to 15, with a median age of 5 years old. We identified age as one of the main drivers in gene expression during malaria infection in this cohort. Parasitemia, the number of parasites in the blood during the time of sampling, was also a main driver of gene expression, and ranged from 48 to 622,775 parasites per microliter of blood, with a median of 26,675. The total number of malaria episodes experienced in 3 years of followup and the time to the next malaria episode after our sequenced blood sample were measured for each child and weighted for the monthly risk of malaria in this seasonal transmission zone (see materials and methods for detailed calculations).
Recruitment	Patient samples used in this study were available at University of Maryland, Baltimore from a previous cohort study from Coulibaly et al. 2014, where consent was given for genetic and genomic analyses. Patients were not directly recruited for our presented study.
Ethics oversight	Institutional review boards of the Faculty of Medicine, Pharmacy and Dentistry of the University of Maryland, Baltimore and of the University of Sciences, Techniques and Technologies of Bamako, Mali (IRB numbers HCR-HP-00041382 and HP-00085882)

Note that full information on the approval of the study protocol must also be provided in the manuscript.

Field-specific reporting

Please select the one below that is the best fit for your research. If you are not sure, read the appropriate sections before making your selection.

☒ Life sciences ☐ Behavioural & social sciences ☐ Ecological, evolutionary & environmental sciences

For a reference copy of the document with all sections, see nature.com/documents/nr-reporting-summary-flat.pdf

Life sciences study design

All studies must disclose on these points even when the disclosure is negative.

Sample size	This study includes transcriptomic analyses from 136 human children during a malaria episode and the parasites causing the episode. These samples were chosen from a previous incidence study described in Coulibaly et al., 2014 and represent one of the largest transcriptomic studies of human malaria infection
Data exclusions	16 samples with fewer than 3 years of available epidemiological data were excluded from analyses of total number of infections and time to next infection, noted in the text and highlighted in Supplemental Table 1.
Replication	This study includes 136 field isolates from a malaria endemic setting. Given the large sample size presented, we expect that these samples represent the wider population of malaria-infected children in this transmission setting in Mali and our results are consistent with those presented in other field studies from similar settings.
Randomization	Randomization was not relevant to this study, as we analyzed gene expression changes as they occur continuously with our chosen covariates, rather than grouping individuals into discrete categories.
Blinding	Blinding was not relevant to this study, as we analyzed gene expression changes as they occur continuously with our chosen covariates, rather than grouping individuals into discrete categories.

Reporting for specific materials, systems and methods

We require information from authors about some types of materials, experimental systems and methods used in many studies. Here, indicate whether each material, system or method listed is relevant to your study. If you are not sure if a list item applies to your research, read the appropriate section before selecting a response.

Materials & experimental systems

n/a	Involved in the study
☒	☐ Antibodies
☒	☐ Eukaryotic cell lines
☒	☐ Palaeontology and archaeology
☒	☐ Animals and other organisms
☒	☐ Clinical data
☒	☐ Dual use research of concern
☒	☐ Plants

Methods

n/a	Involved in the study
☒	☐ ChIP-seq
☒	☐ Flow cytometry
☒	☐ MRI-based neuroimaging

Plants

Seed stocks	*Report on the source of all seed stocks or other plant material used. If applicable, state the seed stock centre and catalogue number. If plant specimens were collected from the field, describe the collection location, date and sampling procedures.*
Novel plant genotypes	*Describe the methods by which all novel plant genotypes were produced. This includes those generated by transgenic approaches, gene editing, chemical/radiation-based mutagenesis and hybridization. For transgenic lines, describe the transformation method, the number of independent lines analyzed and the generation upon which experiments were performed. For gene-edited lines, describe the editor used, the endogenous sequence targeted for editing, the targeting guide RNA sequence (if applicable) and how the editor was applied.*
Authentication	*Describe any authentication procedures for each seed stock used or novel genotype generated. Describe any experiments used to assess the effect of a mutation and, where applicable, how potential secondary effects (e.g. second site T-DNA insertions, mosiacism, off-target gene editing) were examined.*
