# Supplementary material for: Gene expression analyses reveal differences in children’s response to malaria according to their age
Source: Nat Commun. 2024 Mar 6;15:2021. doi: 10.1038/s41467-024-46416-3 (PMC10918175; doi:10.1038/s41467-024-46416-3)
Supplement: Supplementary file 2 — Peer Review File [file 41467_2024_46416_MOESM2_ESM.pdf]

nature portfolio

# Peer Review File

Gene expression analyses reveal differences in children's response to malaria according to their age

**Open Access** This file is licensed under a Creative Commons Attribution 4.0 International License, which permits use, sharing, adaptation, distribution and reproduction in any medium or format, as long as you give appropriate credit to the original author(s) and the source, provide a link to the Creative Commons license, and indicate if changes were made. In the cases where the authors are anonymous, such as is the case for the reports of anonymous peer reviewers, author attribution should be to 'Anonymous Referee' followed by a clear attribution to the source work. The images or other third party material in this file are included in the article's Creative Commons license, unless indicated otherwise in a credit line to the material. If material is not included in the article's Creative Commons license and your intended use is not permitted by statutory regulation or exceeds the permitted use, you will need to obtain permission directly from the copyright holder. To view a copy of this license, visit http://creativecommons.org/licenses/by/4.0/.

Reviewers' Comments:

Reviewer #1:
Remarks to the Author:
Tebben et al. present a study on gene expression in children infected with P. falciparum, the most significant causative agent of malaria in developing countries. Their approach integrates transcriptomics targeting both the infectious agent and the responses of the human host against it ("dual RNAseq"). Specifically, the study centers on the most vulnerable group affected by malaria, namely, young children. The sampling scheme and follow-up data employed in this research are distinctive, offering valuable insights into the interaction between the host and parasite, thereby contributing to understanding of the resultant disease. This study holds considerable interest and relevance within the field.

The first question addressed in the study pertains to the factors influencing variations in overall gene expression. The application of variance partitioning within mixed effect models has been proven highly suitable for addressing this specific question. This method offers a comprehensive overview of the combined response separately for both host and parasite. Additionally, a secondary analysis focused on individual genes (i.e., the quantification of differentially expressed genes) aligns with the overall analysis, providing deeper mechanistic insights. There is potential (as noted in my min or comment below) to further emphasize this aspect, potentially in Table 2.

The subsequent parts of the study employ deconvolution analysis to examine whether variations in immune cell composition (human) or parasite stages (Plasmodium) account for variance in gene expression. The findings indicate that neutrophil and T cell proportions are the primary drivers of human expression differences. Moreover, the authors effectively demonstrate that differences in parasite stages underlie the variations in gene expression observed within the parasite. This analysis is appropriate and very informative.

My ONLY MAJOR COMMENT pertains to the introduction and presentation of the motivation for this analysis:

I find l68-70 (in the introduction): "Specifically, it is often difficult to determine if apparent gene expression differences among samples are i) genuine differences caused by differential gene regulation or, ii) caused by variations, among patients, in the relative proportions of white blood cell subsets or parasite developmental stages." not ideal to reflection the value of the findings. The same is true for similar reasoning in results/discussion e.g. lines 176-184.

In my opinion the identification of different cells associated with expression differences is an asset of the present study. While I understand the quoted text from a cell biology perspective, it could still be reformulate more positively: gene expression differences in bulk RNA sequencing can point to differences in the cell proportions, these are in my view "genuine" and provide very valuable information. The authors discuss nicely that fluorescence activated cell sorting could give similar results, but it might be harder to apply in the field than transcriptomics.

Besides patient age, which induces additional subtle expression differences, the entire remainder of the manuscript is devoted to examining these outcomes. Presenting cell-type differentiation as a primary objective of the study could provide readers with easier access to this pivotal finding. Similarly, the term "corrected for" concerning cell type/parasite stage proportion might inadvertently devalue the significance of the results. I interpret the "correction" as a method to estimate the strength of signal for various cell types—an affirmative aspect in the identification process—rather than implying a scarcity of gene expression differences beyond cell-type deconvolution analyses. Would it not be better to describe cell-type and stage proportions as predictors that were tested, with a very positive outcome?!

Another noteworthy result in the study pertains to the association between child age and gene expression. This finding, considered among the most intriguing outcomes, goes beyond cell types. Notably, however, even for this analysis distinctions in cell types (neutrophils vs. T-cells) and variations in parasite stages (more male gametes observed in older children) are highlighted.

In summary, I perceive the distinctions in host immune-cell and parasite stage proportions as the primary and notably authentic contribution of this study—an aspect that holds significant real-world value in therapeutic, epidemiological, and preventative contexts compared to potential mechanistic differences at the cellular level. Considering the study's focus on cell proportions, I recommend highlighting the deconvolution analysis's capacity to identify cell types as a valuable asset. Additionally, portraying the assessment of deduced cell-types as a predictor variable with substantial explanatory power, rather than merely a "correction," would enhance the delivery of the important results of the study.

I also think that cell-type and parasite stage proportions, as the main result of the study, should be better visualized. Could you derive predictions of proportions (y-axis) e.g. along age or along infection intensity (on a x-axis)? Instead of the single line plots for one cell/stage type (2c/d; 3c/d), stacked area plots along those axes representing all inferred proportions could be informative and highlight the main finding of the study!

Finally, as only POTENTIAL ADDITION to the presented analyses, I'd find co-expression/correlation analysis among host and parasite genes interesting. I'd expect e.g. T-cell (negtatively) and neutrophile (positively) associated genes to correlate with those involved male gametogenesis. Basic co-expression analyses maybe simply performed based on correlation coefficients and/or p-values for among-gene correlations (instead of more involved methods using empirical p-values). It might also be plausible to incorporate cell-type proportions into these correlation analyses to assess whether specific genes or gene clusters (maybe cluster the correlation networks?) exhibit stronger correlations between host and parasite, surpassing the associations seen purely with cell proportions. This addition could potentially enrich the understanding of the interplay between host and parasite genes in this context.

MINOR comments:
Looking at Table 1, the number of subsequent symptomatic malaria episodes (after the first) is given as 0.97 median and range 0 – 2.48. I was confused how is it possible to have a non-integer number as a maximal. I then found the answer in "Weighted number of and time between subsequent infections", as "**" (for months to next infection) points to this. "*" (for subsequent infections) should also point to this (part of the methods).

l159 "Consistent with the results of the analysis of variance presented above, many host and parasite genes []..." Table 2 could visualize this consistency by including the overall variance explained (it currently only has the # of DEGs).

The visual appearance of the "volcano-plots" (Figures 2ab 3ab) might not be most optimal. Information content is cramped into a very small visual space. Maybe you could change aspect ratio? Change the axis limits (different ranges for different plots)? Maybe use "inserts", to zoom into the central "blob" of points and use opacity or 2d-density estimates to avoid an overplotted mass of points?!

l.497 "13 genes were significantly associated [with (!)] this variable [occurrence of subsequent malaria episodes], including three genes that are characteristic of (although not exclusive to) a type 1 IFN (IFN1) response: CXCL10, SOCS1, PLAAT4" -> Would it be possible to give a directionality of this response instead of the somewhat vague "association"? Are elevated Inf-g responses rather predictive of protection or susceptibility?

l598 (also 601) "Models used to estimate the gene expression associated with parasitemia were _adjusted for_ host age and ... " I find this wording a bit odd, these were regular predictor variables, which are tested for significance. They represent own hypotheses being tested not only "adjustments", right? Should these not be called predictors instead of adjustments?

Reviewer #2:
Remarks to the Author:
Peer review of the manuscript entitled "Gene expression analyses reveal differences in children's response to malaria according to their age " by Kieran Tebben et al, submitted for consideration in Nature Communications. This review covers the differential dual expression of humans and P. falciparum genes according to parasitemia and age in the context of symptomatic non-severe P. falciparum malaria infections in Mali.

The authors performed a profiling of host and parasite transcriptomes of 136 whole blood samples from children experiencing symptomatic uncomplicated malaria episodes. I agree this is very interesting to understand the dramatic differences in terms of parasitemia (parasitemia units ranging from 48 – 622,775 in table 1 should include units: parasites/uL?). Yet, the differences in the number of malaria episodes does not seem so dramatic (0 – 2.48, Table 1).

Since malaria in Mali is characterized as being seasonal, and infected people are frequently asymptomatic carriers, I wonder whether the authors had access or even explored the possibility of including in this analysis samples from asymptomatic children from another cohort. This would increase the relevance of this manuscript as gene expression profiles, and especially from human genes, could help shed light into the question regarding why some children are symptomatic and other asymptomatic, apart from their antibody's levels due to repetitive exposure.

Of great importance for the conclusions of this manuscript is the level of parasitemia. The authors report that the percent of reads mapped to the P. falciparum genome was significantly correlated with the parasitemia determined microscopically (p = 6.03 x10 14, r2=0.34). In Supp Fig 1 one can see that at low read numbers this correlation is not so groundbreaking. Do the authors think that this could have influences the results obtained after normalization by parasitemia? The authors should comment of this.

Another way to strengthen this manuscript would be to correlate the gene expression with the total parasite biomass estimates in the form levels of HRP2 or even between as Ptot (Dondoorp et al, PLOS Med 2004) if the authors have access to clinical data regarding levels of HRP2, hematocrit, and weight for its calculation. This would provide a better estimate of total parasite biomass , including sequestered ones, and not only circulating parasitemia.

The authors found two variables contributing to the overall variance in host gene expression: the child's age and parasitemia, explaining on average 3% and 2% respectively of the overall variance in host gene expression. 21 No. Human DEGs and 71 P. falciparum DEGs relied on parasitemia while 1,485 Human DEGs and 6 P. falciparum DEGs relied on children´s age. How many of these genes were common?

The authors explain that they have employed a custom signature matrix derived from P. berghei scRNA-seq data was used for P. falciparum stage deconvolution, using orthologous genes between the two species. I believe it would be of interest to double cross with data from Bozdech et al PLOS Bio 20023 and/or Painter et al NatComms 2018 regarding RNAseq data in vitro.
The authors explain that parasite gene expression associated with parasitemia is primarily driven by differences in stage composition. This fact has already been suggested by Tonkin-Hill et al PLOS Biol 2018 and investigated by Andrade et al in the context of asymptomatic vs symptomatics P. falciparum

infections, NatMed 2020, and more in depth by Thomson-Luque et al in a NatComms 2021 metanalysis. Yet, this is not acknowledged here. They should be references to these publications in this manuscript.

The authors should better explain the at first sight contradictory data regarding gametocyte development genes such as PfNOT1-G, PfFACT-L being not correlated with parasitemia while those involved in gametocytes commitment/gametocytogenesis such as PfCDPK1, PfPfs16 and PfMCA2 being negatively associated.

Finally, the authors comment several times that flow cytometry prior to bulk RNA-seq would be important for higher resolution in several approaches here presented. Although I agree that this is complicated to implement in field setting, I believe that the Malaria Research and Training Center in Bamako has some access to flow cytometry and this work would greatly benefit now or in future studies to this complementary approach.

Reviewer #3:
Remarks to the Author:
This analysis uses whole blood transcriptomics of both the parasite and the host genome in children with a symptomatic infection aged 1-15 years in Mali. Analysis uses deconvolution of both cell composition and parasite development stage to attempt to untangle the associations between age/parasite density and host and parasite transcriptomic pathways. This approach builds on previous similar studies by attempting to take into account more clinical variables with both host and parasite analysis. Key findings significant to the field is that gene expression differences associated with parasitemia were largely driven by changes to host cell composition and parasite development, and that age was a major driver of gene expression profiles. While these results are not surprising, they do highlight the need to account for these confounders in interpreting data in prior studies, and present a nice analysis of age dependent differences in immune responses that may be linked to immunity to malaria.

1. The analysis includes the association of transcriptomic signature on future malaria incidence. Is it also possible to investigate the association of past malaria on transcriptome?
2. In the section on genes associated with protection from future malaria episodes – it is very unclear the direction of association of genes mentioned – I assumed that the type I IFN genes are associated with protection (in blue in Sup Figure 8), but CXCL10 is not listed in this figure? I think that the Sup Table 3 is also miss called – so I found it difficult to match the raw data of this file.
3. A weakness of the data is that no specific hypothesis is further tested/confirmed. Authors do highlight this is required in further studies, and it may be outside of the scope of this current project to do so.

Minor

4. Although the authors account for variation of transmission, it was unclear to me when the samples of each person were collected relative to the transmission season. I suggest this data be more clearly depicted.
5. Some of the Sup figures/tables seem to be miss-called in the text. Check carefully.

We thank the reviewers for their kind assessment of our manuscript and their constructive comments. We have included their suggestions and addressed their concerns in the revised version of this manuscript. We present below detailed answers to all their comments (the page numbers refer to the revised manuscript).

**Reviewer #1**

Tebben et al. present a study on gene expression in children infected with *P. falciparum*, the most significant causative agent of malaria in developing countries. Their approach integrates transcriptomics targeting both the infectious agent and the responses of the human host against it ("dual RNAseq"). Specifically, the study centers on the most vulnerable group affected by malaria, namely, young children. The sampling scheme and follow-up data employed in this research are distinctive, offering valuable insights into the interaction between the host and parasite, thereby contributing to understanding of the resultant disease. This study holds considerable interest and relevance within the field.
The first question addressed in the study pertains to the factors influencing variations in overall gene expression. The application of variance partitioning within mixed effect models has been proven highly suitable for addressing this specific question. This method offers a comprehensive overview of the combined response separately for both host and parasite. Additionally, a secondary analysis focused on individual genes (i.e., the quantification of differentially expressed genes) aligns with the overall analysis, providing deeper mechanistic insights. There is potential (as noted in my minor comment below) to further emphasize this aspect, potentially in Table 2.
The subsequent parts of the study employ deconvolution analysis to examine whether variations in immune cell composition (human) or parasite stages (Plasmodium) account for variance in gene expression. The findings indicate that neutrophil and T cell proportions are the primary drivers of human expression differences. Moreover, the authors effectively demonstrate that differences in parasite stages underlie the variations in gene expression observed within the parasite. This analysis is appropriate and very informative.

We thank the reviewer for the kind assessment of our work and its relevance to the field.

My ONLY MAJOR COMMENT pertains to the introduction and presentation of the motivation for this analysis:

I find l68-70 (in the introduction): "Specifically, it is often difficult to determine if apparent gene expression differences among samples are i) genuine differences caused by differential gene regulation or, ii) caused by variations, among patients, in the relative proportions of white blood cell subsets or parasite developmental stages." not ideal to reflection the value of the findings. The same is true for similar reasoning in results/discussion e.g. lines 176-184.
In my opinion the identification of different cells associated with expression differences is an asset of the present study. While I understand the quoted text from a cell biology perspective, it could still be reformulate more positively: gene expression differences in bulk RNA

sequencing can point to differences in the cell proportions, these are in my view "genuine" and provide very valuable information. The authors discuss nicely that fluorescence activated cell sorting could give similar results, but it might be harder to apply in the field than transcriptomics.
Besides patient age, which induces additional subtle expression differences, the entire remainder of the manuscript is devoted to examining these outcomes. Presenting cell-type differentiation as a primary objective of the study could provide readers with easier access to this pivotal finding.

We thank the reviewer for this suggestion. We have extensively edited the manuscript according to the reviewer's comment: we have rephrased the introduction (line 68-78) and reorganized the result sections to emphasize the value of the analyses of cell proportions.

Similarly, the term "corrected for" concerning cell type/parasite stage proportion might inadvertently devalue the significance of the results. I interpret the "correction" as a method to estimate the strength of signal for various cell types—an affirmative aspect in the identification process—rather than implying a scarcity of gene expression differences beyond cell-type deconvolution analyses. Would it not be better to describe cell-type and stage proportions as predictors that were tested, with a very positive outcome?!
Another noteworthy result in the study pertains to the association between child age and gene expression. This finding, considered among the most intriguing outcomes, goes beyond cell types. Notably, however, even for this analysis distinctions in cell types (neutrophils vs. T-cells) and variations in parasite stages (more male gametes observed in older children) are highlighted.
In summary, I perceive the distinctions in host immune-cell and parasite stage proportions as the primary and notably authentic contribution of this study—an aspect that holds significant real-world value in therapeutic, epidemiological, and preventative contexts compared to potential mechanistic differences at the cellular level. Considering the study's focus on cell proportions, I recommend highlighting the deconvolution analysis's capacity to identify cell types as a valuable asset. Additionally, portraying the assessment of deduced cell-types as a predictor variable with substantial explanatory power, rather than merely a "correction," would enhance the delivery of the important results of the study.

We have thoroughly edited the manuscript to address the reviewer's comment and now present, as suggested, first an analysis of the cell proportion differences and their implications, and second, an examination of the remaining/unexplained gene expression differences. These are now clearly presented as separate analyses rather than just a correction (e.g., lines 182-185 or 302-305). Again, we thank the reviewer for this suggestion that improved our manuscript and emphasized the different aspects of our study's findings.

I also think that cell-type and parasite stage proportions, as the main result of the study, should be better visualized. Could you derive predictions of proportions (y-axis) e.g. along age or along infection intensity (on a x-axis)? Instead of the single line plots for one cell/stage type (2c/d;

3c/d), stacked area plots along those axes representing all inferred proportions could be informative and highlight the main finding of the study!

While the correlations between cell type proportion and age or parasitemia are statistically (and biologically) significant, the small magnitude of the changes and the high amount of inter-individual variations make the stacked bar plots difficult to interpret. We have included an example below of samples ordered by age but would prefer to keep the current displays in our manuscript.

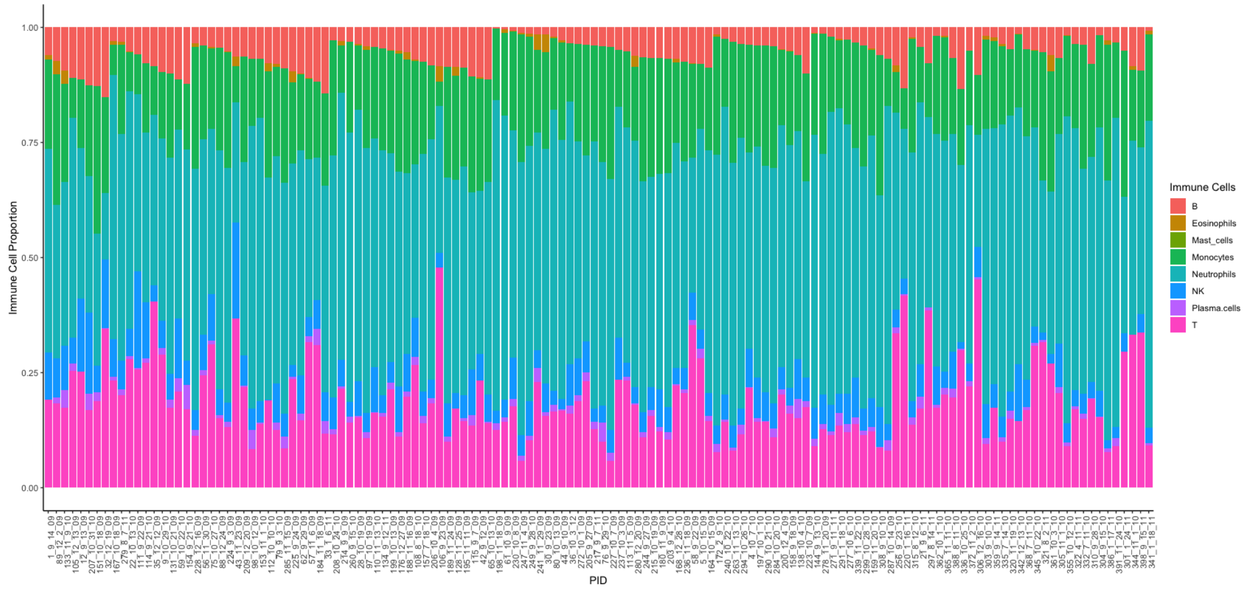


Finally, as only POTENTIAL ADDITION to the presented analyses, I'd find co-expression/correlation analysis among host and parasite genes interesting. I'd expect e.g. T-cell (negtatively) and neutrophile (positively) associated genes to correlate with those involved male gametogenesis. Basic co-expression analyses maybe simply performed based on correlation coefficients and/or p-values for among-gene correlations (instead of more involved methods using empirical p-values). It might also be plausible to incorporate cell-type proportions into these correlation analyses to assess whether specific genes or gene clusters (maybe cluster the correlation networks?) exhibit stronger correlations between host and parasite, surpassing the associations seen purely with cell proportions. This addition could potentially enrich the understanding of the interplay between host and parasite genes in this context.

We attempted the analyses suggested by the reviewer. We used WGCNA to cluster human genes based on their expression across all individuals (to reduce the data dimensionality) before "annotating" them by assessing the correlation between each eigenvector and the proportion of the main white blood cell populations. We then estimated the correlation between the eigenvector of each human gene cluster and the expression of each *P. falciparum* gene. Out of the 2,484 *P. falciparum* genes tested, only 65 genes showed a Pearson's R greater than 0.5 ($R^2$>0.25) with any of the human gene clusters and those were all correlated with the

clusters corresponding to T-cells or Neutrophils (which are themselves correlated with the parasitemia). Due to the high extent of intra-correlation in our dataset, we are not convinced that this analysis is informative or easily interpretable (in our cohort). Unless the reviewer strongly believes that we should include it, we would prefer to focus on the analyses currently presented in the manuscript.

MINOR comments:
Looking at Table 1, the number of subsequent symptomatic malaria episodes (after the first) is given as 0.97 median and range 0 – 2.48. I was confused how is it possible to have a non-integer number as a maximal. I then found the answer in "Weighted number of and time between subsequent infections", as "**" (for months to next infection) points to this. "*" (for subsequent infections) should also point to this (part of the methods).

To address the reviewer's comment (and similar concerns by Reviewer 2), we have modified Table 1 to present the actual numbers of subsequent infection and time to next infections, which are easier to interpret than the normalized values. We discuss the normalization on lines 105-106 and 565-576 and in the footnote in Table 1.

l159 "Consistent with the results of the analysis of variance presented above, many host and parasite genes []..." Table 2 could visualize this consistency by including the overall variance explained (it currently only has the # of DEGs).

We now include those numbers in Table 2 as suggested.

The visual appearance of the "volcano-plots" (Figures 2ab 3ab) might not be most optimal. Information content is cramped into a very small visual space. Maybe you could change aspect ratio? Change the axis limits (different ranges for different plots)? Maybe use "inserts", to zoom into the central "blob" of points and use opacity or 2d-density estimates to avoid an overplotted mass of points?!

We have updated the figures to increase the visibility of the data. We changed the axis limits for each figure to reduce white space (although we chose to keep the limits consistent between A and B for each figure for sake of clarity). We also now highlight on each figure the genes discussed in the text.

l.497 "13 genes were significantly associated [with (!)] this variable [occurrence of subsequent malaria episodes], including three genes that are characteristic of (although not exclusive to) a type 1 IFN (IFN1) response: CXCL10, SOCS1, PLAAT4" -> Would it be possible to give a directionality of this response instead of the somewhat vague "association"? Are elevated Inf-g responses rather predictive of protection or susceptibility?

We have updated the text to reflect directionality and clarify the associations (lines 502-506). Thank you for bringing this issue to our attention.

l598 (also 601) "Models used to estimate the gene expression associated with parasitemia were _adjusted for_ host age and ... " I find this wording a bit odd, these were regular predictor variables, which are tested for significance. They represent own hypotheses being tested not only "adjustments", right? Should these not be called predictors instead of adjustments?

The reviewer is correct. We have updated the text to clarify our model parameters (lines 606-610).

**Reviewer #2**

Peer review of the manuscript entitled "Gene expression analyses reveal differences in children's response to malaria according to their age " by Kieran Tebben et al, submitted for consideration in Nature Communications. This review covers the differential dual expression of humans and P. falciparum genes according to parasitemia and age in the context of symptomatic non-severe P. falciparum malaria infections in Mali.
The authors performed a profiling of host and parasite transcriptomes of 136 whole blood samples from children experiencing symptomatic uncomplicated malaria episodes. I agree this is very interesting to understand the dramatic differences in terms of parasitemia (parasitemia units ranging from 48 – 622,775 in table 1 should include units: parasites/uL?). Yet, the differences in the number of malaria episodes does not seem so dramatic (0 – 2.48, Table 1).

We have updated Table 1 to include units (thank you for noticing this oversight) as well as to replace the normalized values by the actual number of subsequent malaria episodes (see also the response to Reviewer 1).

Since malaria in Mali is characterized as being seasonal, and infected people are frequently asymptomatic carriers, I wonder whether the authors had access or even explored the possibility of including in this analysis samples from asymptomatic children from another cohort. This would increase the relevance of this manuscript as gene expression profiles, and especially from human genes, could help shed light into the question regarding why some children are symptomatic and other asymptomatic, apart from their antibody's levels due to repetitive exposure.

We agree with the reviewer that analyses of asymptomatic individuals would be extremely informative. Unfortunately, we do not have blood samples collected from these participants at baseline or during asymptomatic infections. We are in the process of collecting new blood samples (from a different cohort) to address this limitation in future studies.

Of great importance for the conclusions of this manuscript is the level of parasitemia. The authors report that the percent of reads mapped to the P. falciparum genome was significantly correlated with the parasitemia determined microscopically (p = 6.03 x10 14, r2=0.34). In Supp Fig 1 one can see that at low read numbers this correlation is not so groundbreaking. Do the

authors think that this could have influences the results obtained after normalization by parasitemia? The authors should comment of this.

Supplemental Figure 1 was misleading as only one of the axes was on the logarithmic scale (we have now fixed this issue and replaced Supplemental Figure 1). The correlation is actually quite striking as the percentage of reads mapped *P. falciparum* likely depends on the parasitemia, the stage of the parasite (since some stages are transcriptionally more active than others) and the white blood cell counts. Overall, very few samples had less than 1 million reads mapped to the *P. falciparum* genome (12 out of 136) and the read depth is unlikely to affect the gene expression difference analysis. (Note that we were also very stringent and removed from the analyses all genes expressed at less than 10 counts per million (cpm) in more than 50% of samples). We have clarified these points in lines 597-599.

Another way to strengthen this manuscript would be to correlate the gene expression with the total parasite biomass estimates in the form levels of HRP2 or even between as Ptot (Dondoorp et al, PLOS Med 2004) if the authors have access to clinical data regarding levels of HRP2, hematocrit, and weight for its calculation. This would provide a better estimate of total parasite biomass , including sequestered ones, and not only circulating parasitemia.

Again, we agree with the reviewer and would have loved to include HRP2 levels in our analyses but, unfortunately, do not have access to serum samples or HRP2 levels from this cohort.

The authors found two variables contributing to the overall variance in host gene expression: the child's age and parasitemia, explaining on average 3% and 2% respectively of the overall variance in host gene expression. 21 No. Human DEGs and 71 P. falciparum DEGs relied on parasitemia while 1,485 Human DEGs and 6 P. falciparum DEGs relied on children´s age. How many of these genes were common?

For both human and *P. falciparum* DEGs, 0 genes overlapped between these two comparisons. This has been added to the text in lines 378-379 and lines 484-485.

The authors explain that they have employed a custom signature matrix derived from P. berghei scRNA-seq data was used for P. falciparum stage deconvolution, using orthologous genes between the two species. I believe it would be of interest to double cross with data from Bozdech et al PLOS Bio 20023 and/or Painter et al NatComms 2018 regarding RNAseq data in vitro.

As noted by the reviewer, estimating the parasite developmental age is a powerful method of correcting for stage differences among *in vitro* samples but this approach might not accurately capture the heterogeneity of *in vivo* samples. Using developmental age to account for differences in stage composition works very well when parasites in different samples only differs by their average developmental age (as is typically the case for synchronized cultures of *P. falciparum* for which this method was developed). *In vivo*, the stage heterogeneity is likely to be more complex and the parasites less "synchronized" (i.e., a single "average age" might not capture the whole

extent of heterogeneity). Even if most asexual parasites in blood are likely to be ring-stage (due to sequestration of mature parasites), small proportions of circulating mature stages could dramatically impact the transcriptional profiles (since they are much more transcriptionally active) and thus introduce extensive variation among samples. In addition, and perhaps more importantly, gametocytes are not accounted for using developmental age and gametocytemia can significantly vary among patient infections. Note also that the custom signature matrix and its use and robustness for deconvoluting *P. falciparum* data has been extensively tested (PMID: 35862820).

The authors explain that parasite gene expression associated with parasitemia is primarily driven by differences in stage composition. This fact has already been suggested by Tonkin-Hill et al PLOS Biol 2018 and investigated by Andrade et al in the context of asymptomatic vs symptomatics P. falciparum infections, NatMed 2020, and more in depth by Thomson-Luque et al in a NatComms 2021 metanalysis. Yet, this is not acknowledged here. They should be references to these publications in this manuscript.

We have incorporated references to these publications within our manuscript (refs 61-63). Thank you for these suggestions.

The authors should better explain the at first sight contradictory data regarding gametocyte development genes such as PfNOT1-G, PfFACT-L being not correlated with parasitemia while those involved in gametocytes commitment/gametocytogenesis such as PfCDPK1, PfPfs16 and PfMCA2 being negatively associated.

We agree with the reviewer and have removed this discussion from the manuscript.

Finally, the authors comment several times that flow cytometry prior to bulk RNA-seq would be important for higher resolution in several approaches here presented. Although I agree that this is complicated to implement in field setting, I believe that the Malaria Research and Training Center in Bamako has some access to flow cytometry and this work would greatly benefit now or in future studies to this complementary approach.

The samples included in our cohort were collected from 2009-2013 and flow cytometry was not performed on these samples at that time, nor were PBMC samples collected for future use. We agree with the reviewer that future studies would benefit from flow cytometry analyses and refer to such studies in our manuscript (although, as noted by Reviewer 1, these analyses are difficult to implement on large numbers of field samples). Of note, our method of gene expression deconvolution, CIBERSORTx, has been benchmarked against flow cytometry and produces quite accurate estimates of the broad cell types we included here.

**Reviewer #3 (Remarks to the Author):**

This analysis uses whole blood transcriptomics of both the parasite and the host genome in

children with a symptomatic infection aged 1-15 years in Mali. Analysis uses deconvolution of both cell composition and parasite development stage to attempt to untangle the associations between age/parasite density and host and parasite transcriptomic pathways. This approach builds on previous similar studies by attempting to take into account more clinical variables with both host and parasite analysis. Key findings significant to the field is that gene expression differences associated with parasitemia were largely driven by changes to host cell composition and parasite development, and that age was a major driver of gene expression profiles. While these results are not surprising, they do highlight the need to account for these confounders in interpreting data in prior studies, and present a nice analysis of age dependent differences in immune responses that may be linked to immunity to malaria.

1. The analysis includes the association of transcriptomic signature on future malaria incidence. Is it also possible to investigate the association of past malaria on transcriptome?

We agree with the reviewer that association with past malaria would be fascinating to study but, unfortunately, we do not have any record or information on past malaria in this cohort. (Note that many of the participants included are very young and might not have had many previous exposures to malaria parasites).

2. In the section on genes associated with protection from future malaria episodes – it is very unclear the direction of association of genes mentioned – I assumed that the type I IFN genes are associated with protection (in blue in Sup Figure 8), but CXCL10 is not listed in this figure? I think that the Sup Table 3 is also miss called – so I found it difficult to match the raw data of this file.

We have updated the text to clarify the direction of associations of these genes (lines 502-505). We have updated supplemental figure 8 to include the label for CXCL10. Supplemental Table 3 should have been a multi-sheet excel document with one sheet containing the statistics for each gene associated with each clinical variable but might have been lost or truncated during formatting. All supplemental tables presented in our manuscript are now also available on our github page to facilitate the review: https://github.com/tebbenk/symptomatic_malaria.

3. A weakness of the data is that no specific hypothesis is further tested/confirmed. Authors do highlight this is required in further studies, and it may be outside of the scope of this current project to do so.

The hypothesis driving this work was centered on the ability to identify transcriptional profiles related to clinical variables. We believe this also provides an important opportunity for hypothesis generation for more targeted molecular work.

Minor

4. Although the authors account for variation of transmission, it was unclear to me when the

samples of each person were collected relative to the transmission season. I suggest this data be more clearly depicted.

The date of sample collection is now included in Supplemental Table 1.

5. Some of the Sup figures/tables seem to be miss-called in the text. Check carefully.

We have carefully checked all references to supplemental figures and tables in the text.

Reviewers' Comments:

Reviewer #1:
Remarks to the Author:
The authors addressed my main comment regarding the framing of the study by reformulating relevant text sections in the manuscript. In my eyes this improves the narrative and presentation of the main results obtained regarding cell proportions. My initial assessment was already positive and these slight changes in the framing and presentation are fully sufficient to make me recommend to accept the manuscript for publication.

Two minor suggestions I had were addressed (only) in the response to reviewers:

- Showing cell-proportions as stacked bar charts:
I agree that it might be difficult to conceive a figure in this style efficiently pointing to the main findings of the manuscript. This is not a failure of the authors but simply follows from the fact that proportions (i.e. differences in small proportions between samples) are hard to visualize. One idea I had looking at the figure in the reviewer response was to try to use z-score normalization across samples... but this might work or not to produce an telling display and might be hard to explain to readers not used to such normalization. Whether they want to try this should be left to the authors. I agree that the presentation in Figure 4 b-e works well!

- Correlation analysis between individual genes across host and parasite: my interest in such an approach was motivated by findings in Mukherjee et al 2021 (https://doi.org/10.1128/msystems.00182). We found in this study that human-falciparum co-expression from two studies was aberrant compared to human-vivax or (orthologue) co-expression in malaria model systems. We were unsure whether this is due to technical limitation of transcriptomics in patient blood or a biological signal of falciparum being phylogenetically different to the other plasmodium species. The brief report in the answers to my reviewer comment seems to indicate that little co-expression was detected in the current study. The approach the authors used (first clustering within the host expression then correlating expression of host clusters to parasite expression) is quite different from the approach in Mukherjee et al and interesting. In the authors response-analysis many host genes (those in within-species clusters) seem to "cluster around" few parasite genes. This might point to similarities with our findings from human-falciparum compared to other malaria (model) systems: lower number of co-expressed genes but also commonalities on a higher functional level as many "neutrophile genes" cluster round few parasite genes.
In the framework of the study such co-expression approaches provide only an additional perspective. The usefulness of this perspective might be quite specific to my interests in host-parasites co-expression. I thank the authors for providing the additional analysis. They should feel free to ignore this for reporting within the manuscript (as they currently do). I agree that the current analyses and presentation are more in line with the overall narrative.

I am happy to have been involved in the publication process of an insightful study and recommend to accept the revised version.

Emanuel Heitlinger

Reviewer #2:
Remarks to the Author:
The authors have satisfactorily answered to my comments. I still missed an least attempting an alternative approach for stage determination such as the one presented by Bozdech et al PLOS Bio 20023 and/or Painter et al NatComms 2018 regarding RNAseq data in vitro which was previous

employed for field isolates in other publications such as in RThomson-Luque et al NatComms 2021.

Thank you for handling our manuscript **"Gene expression analyses reveal differences in children's response to malaria according to their age"** (NCOMMS-23-52305) for its review at *Nature Communications,* and for providing us with constructive and insightful reviews.

Regarding the last suggestions from the reviewers:

- "Showing cell-proportions as stacked bar charts using z-score normalization across samples" We did generate the z-normalized figure as suggested but the result was not very convincing. As the reviewer thought, the figure remains very difficult to read due to the large number of samples and would not be easily interpreted by readers not used to such normalization. We would therefore prefer to keep the current representations of the data.
- "higher reproducibility by improving the README of the repository"

We have significantly improved the readme file on github and it should now provide the information necessary to replicate all the analyses described in the manuscript.

**"I** still missed an least attempting an alternative approach for stage determination such as the one presented by Bozdech et al PLOS Bio .20023 and/or Painter et al NatComms 2018 regarding RNAseq data in vitro which was previous employed for field isolates in other publications such as in RThomson-Luque et al NatComms 2021."

As we discussed in our previous response to the reviewer, this approach only corrects for the average developmental age of asexual parasites and is not designed for *in vivo* samples where asexual parasites are not synchronized and a significant proportion of the parasites are gametocytes. We would therefore prefer not to implement this analysis.

We have addressed all editorial requests from the checklist, including revising the abstract for length and tense and modifying the figure legends to provide additional statistical details.

We hope that you will find our manuscript suitable for publication,

Thank you again for your help with this manuscript,
